# Supplementary material for: Negative allosteric modulators of metabotropic glutamate receptor 3 target the stem-like phenotype of glioblastoma
Source: Mol Ther Oncolytics. 2020 Dec 25;20:166–74. doi: 10.1016/j.omto.2020.12.009 (PMC7851500; doi:10.1016/j.omto.2020.12.009)
Supplement: Document S1. Supplemental Materials and Methods, Notes S1 and S2, Figures S1–S3, and Table S1 [file mmc1.pdf]

OMTO, Volume *20*

Supplemental Information

Negative allosteric modulators of metabotropic glutamate receptor 3 target the stem-like phenotype of glioblastoma

Hans-Georg Wirsching, Manuela Silginer, Elisa Ventura, Will Macnair, Isabel Burghardt, Manfred Claassen, Silvia Gatti, Jürgen Wichmann, Claus Riemer, Hannah Schneider, and Michael Weller

SUPPLEMENTARY MATERIAL

Supplementary Methods

Single-cell RT-PCR

Glioblastoma tissues were obtained from the operating room and immediately dissociated using a papain-based dissociation system (Worthington). Leukocytes were depleted using anti-human CD45 microbeads (Miltenyi Biotec). Cells were magnetically labeled with microbeads labeled with anti-human CD45 antibody for 15 minutes at +4°C. CD45+ and CD45- cells were separated using MACS LD columns (Miltenyi Biotec). Cells were captured on a C1 Single-Preamp IFC (10-17 µm) using the C1 Single-Cell Autoprep instrument (Fluidigm). The C1 Single-Preamp IFC was imaged under an inverted microscope to identify and exclude empty sites or sites with multiple cells or debris. After the generation of preamplified cDNA using the Single Cells-to-CT kit (Life Technologies), pooled primers and Fluidigm STA reagents, qPCR was performed by using the BioMark HD system with IFC Controller HX (Fluidigm) and 2X ssoFAST EvaGreen Supermix with Low Rox (Bio-Rad). Data were collected using the Fludigim Data Collection software. Quality controls included cDNAs derived from a glioblastoma tissue before and after CD45+ depletion and the qPCR Human Reference cDNA, random-primer (Clontech). All primers were tested for amplification efficiency of at least 90% by generating standard curves for each gene. Data was processed via removal of cells with low overall expression values, identification of limits of detection for each analyzed gene and cell-to-cell median normalization.[1]

The following primers were utilized:

mGlu2 fwd 5'-CACGGCAGTGTGTACCTTACG -3’

mGlu2 rv: 5'- GATGCGTGCAATGCGGTTG -3'

mGlu3 fwd: 5'- AGCAATCACTGGAGTTTGTCAG -3'

mGlu3 rv: 5'- GCAATGAGAAGTGGGATGTTTTC -3'

Oct-4 fwd: 5'- CGAGAAGGATGTGGTCCGAG -3'

Oct-4 rv: 5'- TGTGCATAGTCGCTGCTTGA -3'

Nanog fwd: 5'- GAAATACCTCAGCCTCCAGC -3'

Nanog rv: 5'-GCGTCACACCATTGCTATTC -3'

Musashi fwd: 5'- CCAATGGGTACCACTGAAGC -3'

Musashi rv: 5'- ACTCGTGGTCCTCAGTCAGC -3'

Olig2 fwd: 5'- AGCTCCTCAAATCGCATCC -3'

Olig2 rv: 5'- ATAGTCGTCGCAGCTTTCG -3'

GalC fwd: 5'- GGATCTTACAGGGTTACAGGTGA -3'

GalC rv: 5'- TGCTGTAACTTCAACACGTCCT -3'

Tubb3 fwd: 5'- TCTTCTCACAAGTACGTGCCTC -3'

Tubb3 rv: 5'- GTGTAGTGACCCTTGGCCC -3'

Map2 fwd: 5'- TGCCTCAGAACAGACTGTCAC -3'

Map2 rv: 5'- GGCTCTTGGTTACTCCGTCA -3'

SOX2 fwd: 5'- CACACTGCCCCTCTCAC -3'

SOX2 rv: 5'- TCCATGCTGTTTCTTACTCTC -3'

SOX4 fwd: 5'- GGTCTCTAGTTCTTGCACGCT -3’

SOX4 rv: 5'- CTGCAAGAAGGGAGCTGGTAA -3'

KLF4 fwd: 5’- CTCATGCCACCCGGTTCC -3’

KLF4 rv: 5’- TTTCTCACCTGTGTGGGTTCG -3’

PDGFA fwd: 5’- CGTAGGGAGTGAGGATTCTTTGG -3’

PDGFA rv: 5’- AATGACCGTCCTGGTCTTGC -3’

L1CAM fwd: 5’- CCCCAGGTCACTATCGGCTA -3’

L1CAM rv: 5’- GACTGCCCTCCCTCCAGTA -3’

MGMT fwd: 5’- GTCGTTCACCAGACAGGTGTT -3’

MGMT rv: 5’- ACAGGATTGCCTCTCATTGCTC -3’.
CD133 fwd: 5'- TGGATGCAGAACTTGACAACGT -3'
CD133 rv: 5'-ATACCTGCTACGACAGTCGTGGT-3'
Arf1 fwd: 5’-GACCACGATCCTCTACAAGC -3’
Arf1 rv: 5’- TCCCACACAGTGAAGCTGATG -3’.

CD133 magnetic-activated cell sorting and RT-PCR

Dissociated tumor cells were consecutively labeled with CD45 and CD133 magnetic MicroBeads at 4°C for 30 minutes each. Depletion of $CD45^+$ cells was followed by separation of $CD133^+$ and $CD133^-$ cell populations utilizing MACS LS columns (Miltenyi Biotec). All differential mRNA expression data were obtained from cells lysed immediately following magnetic sorting. Total RNA for RT-PCR was prepared using the NucleoSpin System (Macherey-Nagel) and complementary DNA transcribed using SuperScript II reverse transcriptase (Invitrogen). Complementary DNA amplification was monitored using SYBR Green Master Mix (Applichem) on the 7300 Real time PCR System (Applied Biosystems). The conditions for these PCR reactions were: 40 cycles of 95°C for 15 s, 60°C for 1 minute. The proper separation of $CD133^+$ and $CD133^-$ cells was confirmed by analyzing CD133 mRNA levels in the two cell populations. Arf1 transcript levels were used as a house-keeping reference for relative quantification of messenger RNA expression levels using the ΔCT method.

Flow cytometry

For detection of Grm3 and CD44 cell surface protein levels, GSC were separated mechanically and with accutase (Gibco Life Technologies, Paisley, UK) and incubated with anti-Grm3-Alexa647 antibody (1:100, bs-12012R-A647, BioSS/Lucerna, Lucerne, Switzerland), anti-CD44-FITC antibody (1:100, 11-0441-82, eBioscience, Vienna, Austria) or respective isotype controls, diluted in phosphate-buffered saline (PBS) containing 0.5% bovine serum albumin, 2 mM EDTA and 1 mM $MgCl_2$. Protein levels were determined using a CyAn flow cytometer (Beckman Coulter).

Immunohistochemistry

Frozen brains of pre-randomized mice embedded in cryomoulds in Shandon Cytochrome yellow (Thermo Scientific, Waltham, MA) were cut into 8 µM sections, fixed with 4 % paraformaldehyde and blocked with blocking solution (Biosystems, Muttenz, Switzerland). Sections were stained with primary antibodies to caspase-3 (#9662; Cell Signaling), cleaved caspase-3 (ASP175, #9661; Cell Signaling), Grm3 (1:200, bs-12012R, BioSS/Lucerna, Lucerne, Switzerland), Oct4 (1:200, ab184665, Abcam, Cambridge, UK) or Sox2 (1:300, ab171380, Abcam, Cambridge, UK) overnight at 4°C. Visualization was done using the SignalStain® Boost IHC Detection Reagent (HRP, Rabbit) (#8114; Cell Signaling) and the ImmPact DAB kit (#SK-4105, Vector Laboratories, Burlingame, CA).

Cyclic adenosine monophosphate assay

A cell-based competitive immunosorbent assay kit to detect cAMP was utilized (Cisbio). In brief, 5,000 LN-229 cells were washed twice with PBS without calcium or magnesium prior to adenyl cyclase stimulation with forskolin at 10 μM and addition of the Grm2/3 agonist LY-379268 at 100 nM or the Grm2/3 allosteric inhibitor RO1 or both. After 15 minutes the reaction was stopped by adding lysis buffer and detection reagents to initiate fluorescence resonance energy transfer (FRET) between XL665-labelled cAMP and a cryptate-labelled monoclonal anti-cAMP antibody. Fluorescent signals were detected at 620 nm and 665 nm, respectively, upon stimulation at 320 nm utilizing a Tecan infinite plate reader (Tecan, Maennedorf, Switzerland). Intercellular cAMP levels were calculated based on the reduction of FRET due to competition of cellular cAMP with XL665-labelled cAMP.

Immunoblot analysis

Denatured whole protein lysates (20 µg/lane) were separated on 10% acrylamide gels. After transfer to nitrocellulose (Bio-Rad), blots were blocked in PBS containing 5% skimmed milk and 0.05% Tween 20 and incubated overnight at 4°C with an anti-pERK antibody (Santa Cruz Biotechnology), washed in PBS and incubated for 1 h at room temperature with a horseradish peroxidase-coupled secondary antibody (Santa

Cruz Biotechnology) and the enhanced chemiluminescence technique (Thermo Fisher Scientific).

Clonogenic growth and sphere formation assays

For clonogenic growth assays of non-GSC, 500 cells per well were seeded in 6-well plates in triplicates overnight and exposed to temozolomide (TMZ) at the individual EC_{50} of each cell line [2] and/or to RO1 or RO2 at 100 nM for 24 h. After 21 days, colonies of at least 50 cells were counted upon staining with crystal violet. Sphere formation of GSC was assessed by separating neurospheres mechanically and with accutase, seeding overnight prior to treatment with RO1 or RO2 at indicated concentrations for 24 hours prior to seeding at 500 cells in 2 ml serum-free neurobasal medium supplemented with growth factors in uncoated culture dishes. Spheres of at least 50 cells were counted after 21 days. Susceptibility of clonogenic growth of GSC to co-treatment with RO1 or RO2 and TMZ was assessed by seeding 100 cells per well in 96-well plates and assessing cell viability after 7-14 days utilizing the MTT assay.

Note S1A

Glioblastoma gene expression subtypes were termed proneural, classical, mesenchymal and neural based on similarities with established gene set expression patterns.[3] The neural subtype reflects mostly normal brain expression patterns in samples with low tumor cell content, e.g. in the infiltration zone of tumors.[4, 5] Proneural glioblastomas comprise a sub-group of tumors that harbor distinct point mutations in the genes encoding isocitrate dehydrogenase (*IDH*)-1 or -2.[3] Patients with *IDH* mutated glioblastomas are younger and experience a relatively more favourable outcome.[6] Therefore, we considered that imbalances in *GRM3* gene expression between *IDH* mutated and *IDH* wild type proneural glioblastomas may explain the association of *GRM3* expression levels with survival. *GRM3* gene expression was balanced between both groups (t-test p=0.93, Fig. S1D) and the transcriptional subtypes do not differ in terms of survival,[3] including when analysis is restricted to patients with IDH wild-type glioblastoma.[4] At an alpha level of 0.1, the association of *GRM3* expression levels with survival was also apparent in the proneural group when patients with IDH mutated glioblastoma were excluded (Fig. S1E, p=0.089). No association of *GRM3* gene expression levels with OS was detected in IDH mutated proneural glioblastomas, albeit the small number of only 9 patients per group precludes definite conclusions (Fig. S1F, p=0.78). No association of high versus low *GRM3* expression levels with overall survival was present in patients with lower-grade gliomas (not shown, log rank p=0.41), which comprised mostly IDH mutated astrocytomas and oligodendrogliomas.[7]

Note S1B

We considered chromosome 7 amplification a likely explanation for high Grm3 expression, because chromosome 7 gain is frequent in IDH wild-type glioblastoma and underlies the upregulation of several oncogenes.[6, 8-10] Indeed, copy number gains of the Grms located on chromosome 7, i.e. *GRM3* and *GRM8*, are present in >75% of glioblastoma samples (Fig. S1G), but *GRM3* copy number gains appear not to be associated with gene expression (Fig. S1H). Therefore, we next interrogated gene methylation patterns of *GRM* genes. The gene encoding Grm3 was the least methylated among all Grm subtypes (Fig. S1I). Overall, the mechanism of differential regulation of *GRM3* remains elusive.

Supplementary Figures

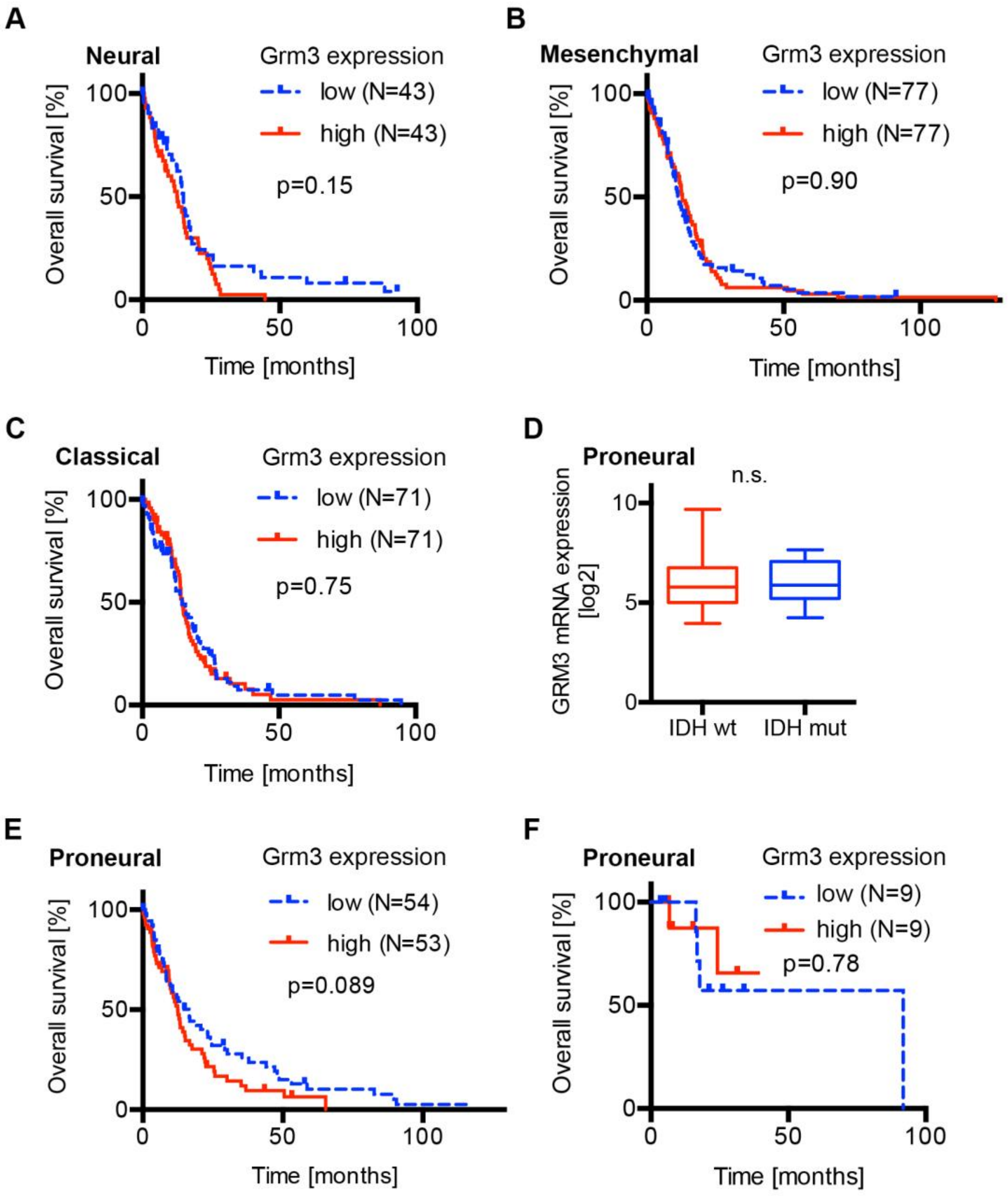

(Figure S1, continued on next page)

(Figure S1, continued)

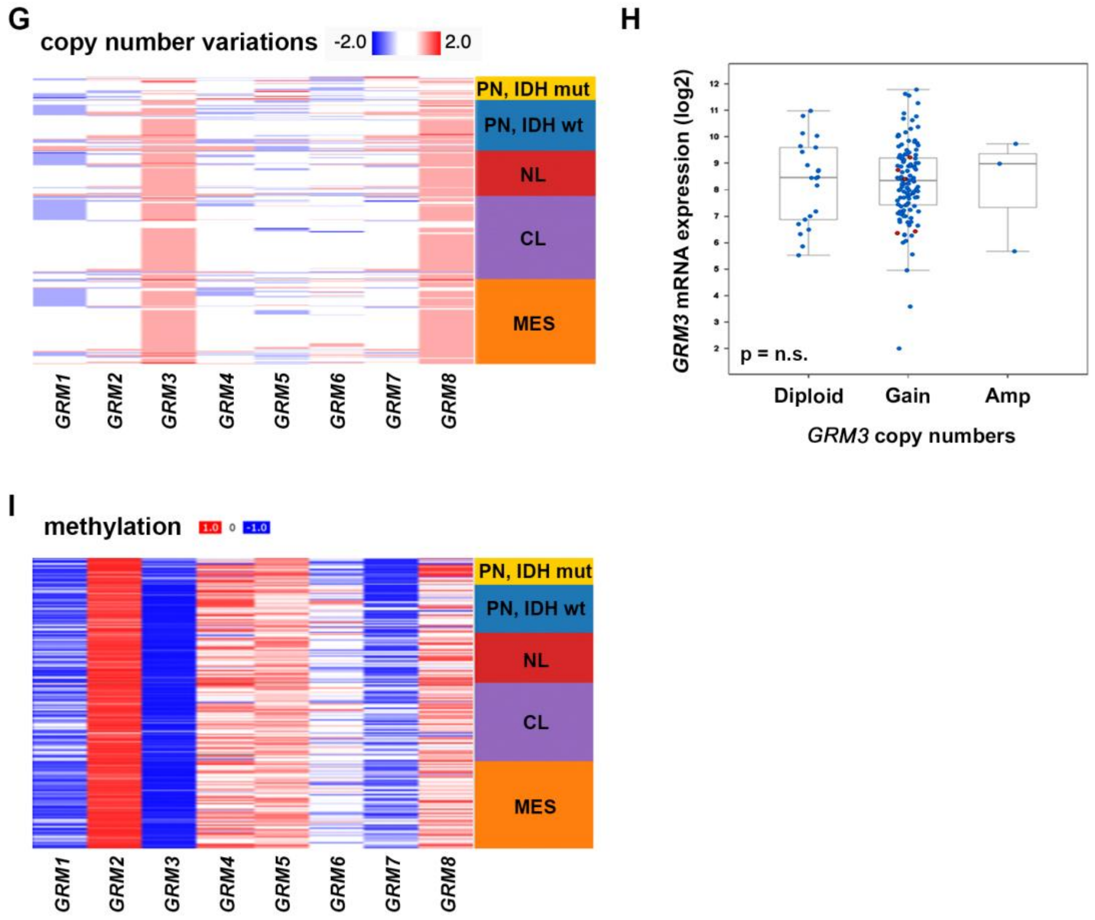

Figure S1. Outcome and molecular analyses of the TCGA glioblastoma dataset. *A-C.* Kaplan Meier curve of patients with indicated glioblastoma gene expression subtypes, stratified by *GRM3* gene expression levels (cut-off: median *GRM3* gene expression). *D. GRM3* gene expression in proneural glioblastoma with IDH wild-type (N=105) versus mutant (N=18); Boxplots represent median and interquartile range, whiskers represent the range; n.s., not significant. *E,F.* overall survival stratified by low versus high *GRM3* gene expression (cut-off: median) in patients with proneural glioblastoma with wild-type *(E)* and mutant *(F)* IDH. *G.* Copy number variations of *GRM* genes by gene expression subtype. Copy numbers were determined by GISTIC (thresholded) utilizing the Xena webtool.[11] *H. GRM3* mRNA expression by copy number. Boxplots represent median and interquartile range (n.s., not significant). *I.* Gene methylation patterns of *GRM* genes (N=288).

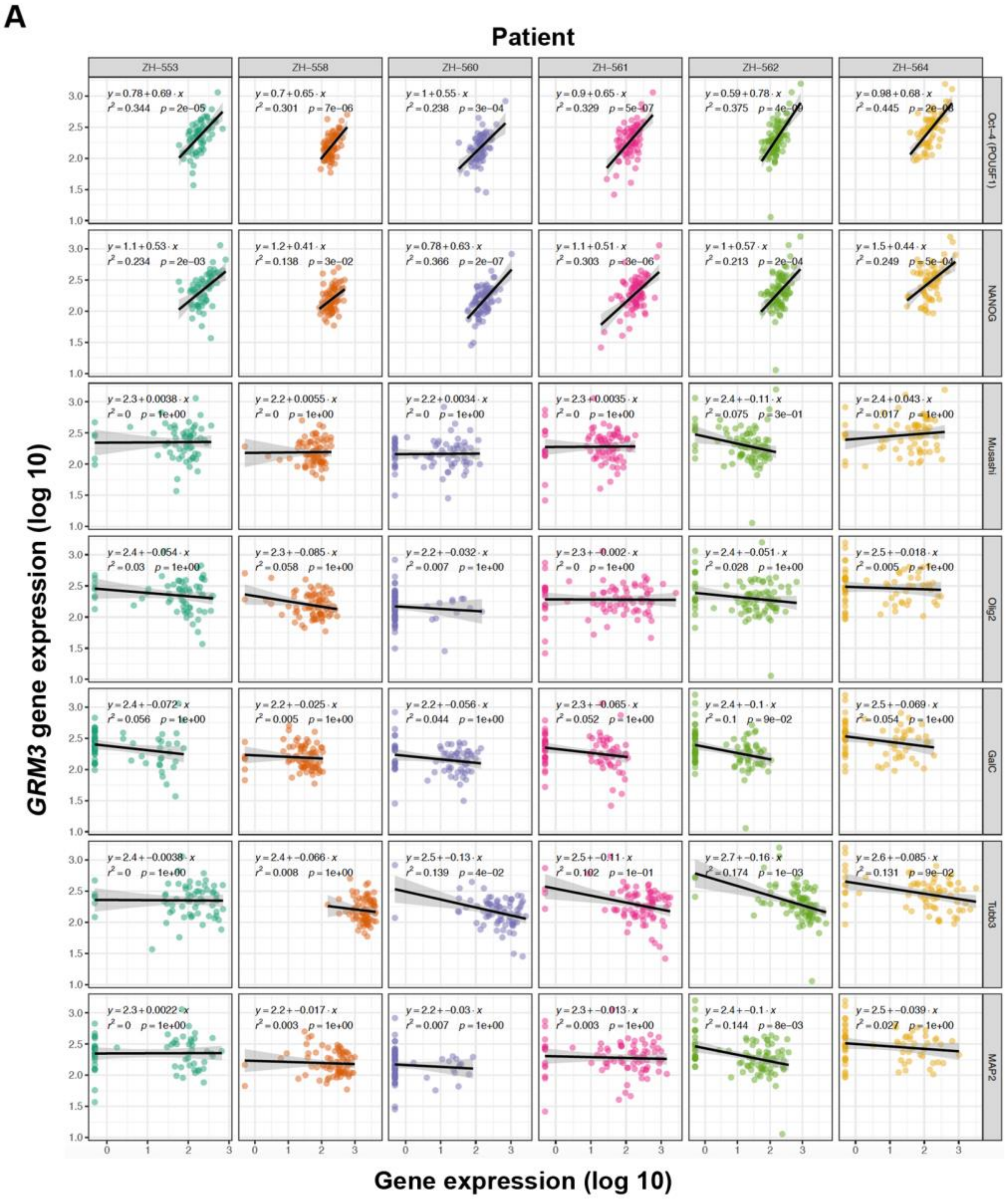

(Figure S2, continued on next page)

(Figure S2, continued)

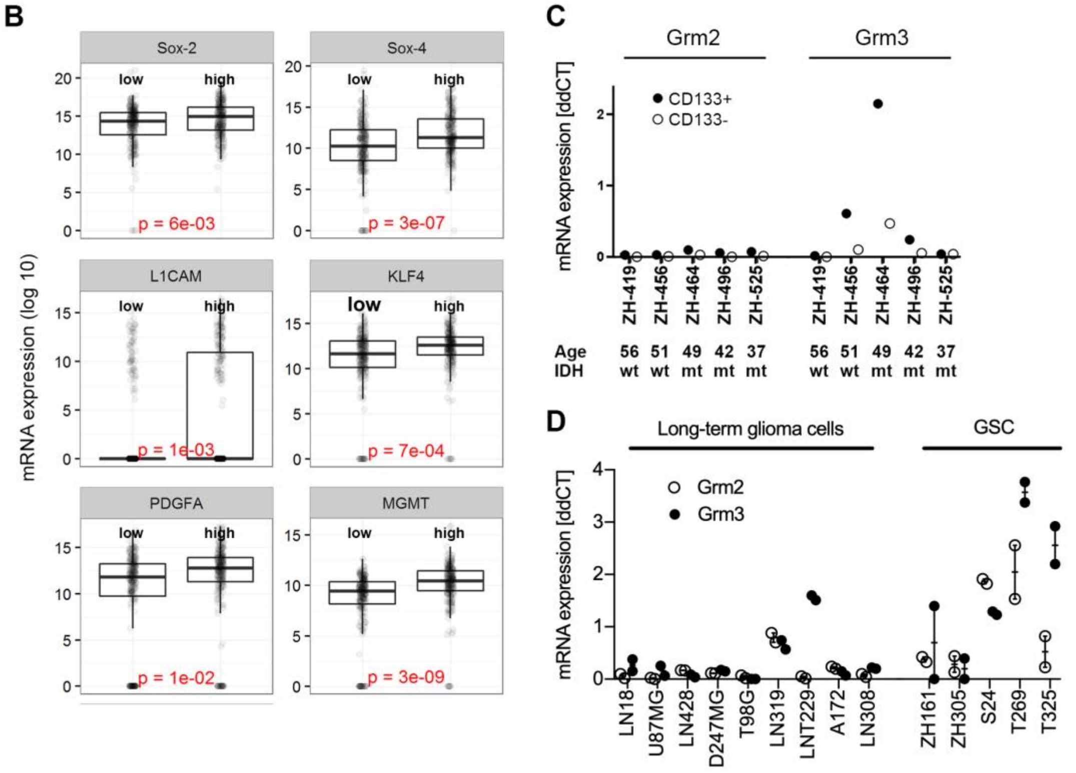

Fig. S2. Grm3 expression in single cells, GSC and cell line models. *A*, *B.* Single cell gene expression analysis in freshly dissected glioblastoma samples (N=6). Gene expression values of individual cells were obtained by scRT-PCR and cell-to-cell median normalization. *A.* Co-expression of Grm3 and neuroglial lineage markers in individual glioblastoma cells *ex vivo*. Individual patients are color-coded and referenced in the top line. Grm3 gene expression is plotted on the y-axis, genes analysed are indicated on the right and plotted on the x-axis. Slopes indicate correlations. Black line indicates a linear regression fitted to the data in each panel; grey area shows 95% confidence interval of this fit; text shows the formula of the fitted linear regression, the R^2 value and the Bonferroni-adjusted p-value for the fit. *B.* Gene expression in single glioblastoma cells with low versus high Grm3 expression. Data from individual patients were pooled (N=6) and cells were stratified by low versus high *GRM3* gene expression (N=482, cut-off: median). Boxes depict the median and interquartile ranges, whiskers extend to the largest value no further than 1.5-fold interquartile ranges from the hinge. Two-sided t-test and Bonferroni adjustment for multiple testing was performed to compare gene expression in low versus high Grm3 expressing cells. *C.* RT-PCR of Grm2 and Grm3 mRNA levels in freshly dissected tumor tissue (N=5) sorted for CD133. *D. GRM2* and *GRM3* gene expression in a panel of long-term glioma cell lines and GSC (mean and SEM, n=2).

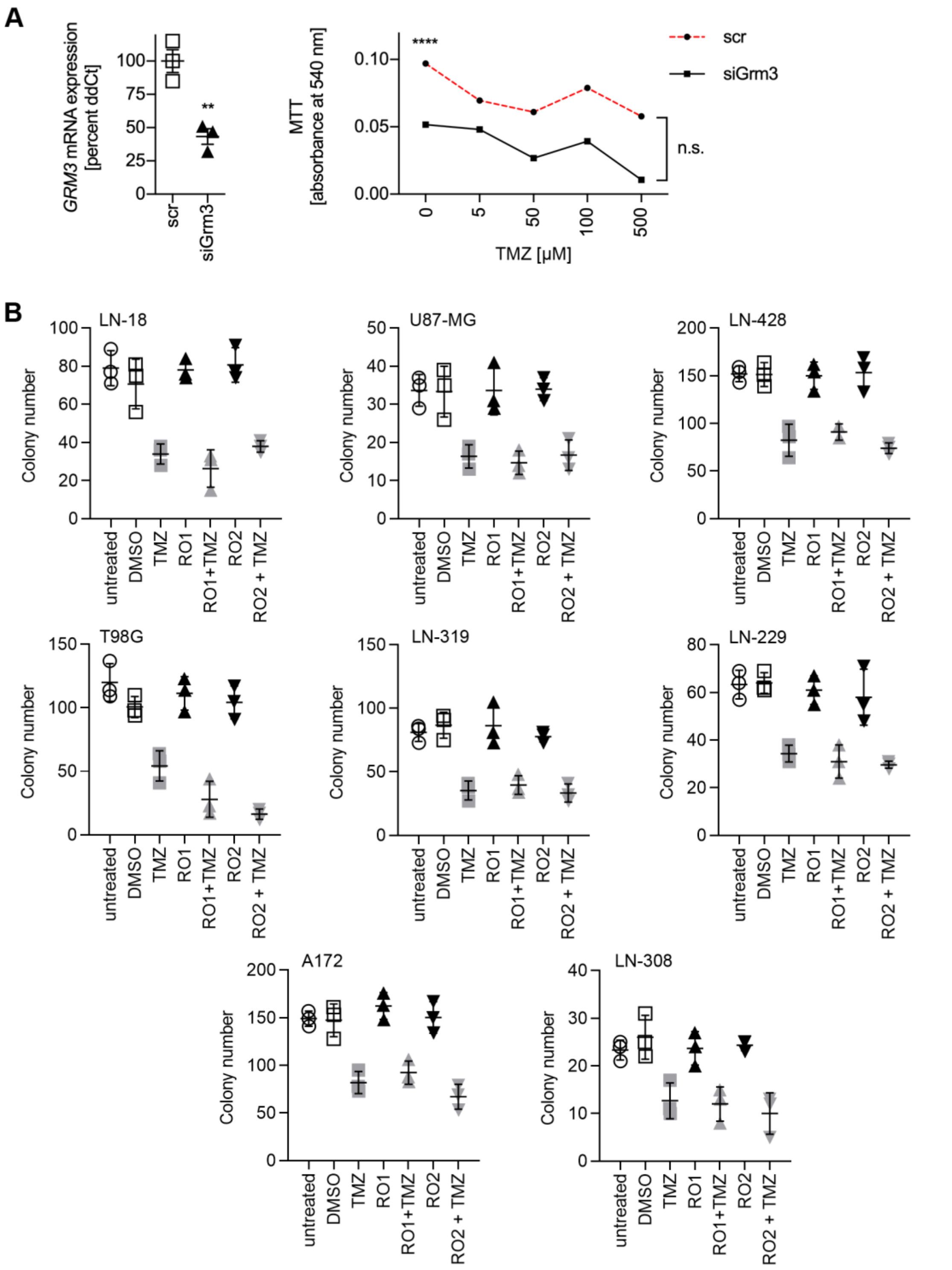

Fig. S3. Inhibition of Grm3 signalling in non-GSC does not affect clonogenic growth or sensitivity to temozolomide (TMZ). *A.* 72 h after transfection of T-325 cells with *GRM3*-targeted siRNA, gene silencing was confirmed by qRT-PCR (left panel), and 100 T-325 cells per well were seeded in 96-well plates in 6 technical replicates and metabolic activity was assessed utilizing the MTT assay on day 7 **** $p<0.001$, t-test corrected for multiple testing utilizing the Holms-Sidak method, error bars: SEM). *B.* Indicated cell lines were seeded at clonal density (500 cells per well in 6-well plates) and treated overnight with TMZ at

the individual EC_{50} of each cell line [2], Grm3 signalling inhibitors (RO1, RO2) at 100 nM, or both. Dimethylsulfoxide (DMSO) was utilized as solvent control. Colony numbers were counted after 21 days. 2-sided t-tests comparing between RO1 or RO2 versus DMSO and RO1+TMZ or RO2+TMZ versus TMZ were not significant for any cell line tested.

Table S1. Clinical and molecular characteristics of patients' samples utilized for single cell RT-PCR.

Patient ID	Number of cells	Age/gender	Diagnosis	MGMT	EGFRvIII
ZH-553	69	63/f	Glioblastoma, IDH wildtype	methylated	yes
ZH-558	82	29/f	Glioblastoma, IDH wildtype	not methylated	No
ZH-560	78	54/m	Glioblastoma, IDH wildtype	not methylated	No
ZH-561	84	33/f	Glioblastoma, IDH wildtype	methylated	Yes
ZH-562	94	82/m	Glioblastoma, IDH wildtype	not methylated	No
ZH-564	75	54/m	Glioblastoma, IDH wildtype	not methylated	No

Supplementary References

1. Livak, KJ, Wills, QF, Tipping, AJ, Datta, K, Mittal, R, Goldson, AJ*, et al.* (2013). Methods for qPCR gene expression profiling applied to 1440 lymphoblastoid single cells. *Methods* **59**: 71-79.

2. Hermisson, M, Klumpp, A, Wick, W, Wischhusen, J, Nagel, G, Roos, W*, et al.* (2006). O6-methylguanine DNA methyltransferase and p53 status predict temozolomide sensitivity in human malignant glioma cells. *J Neurochem* **96**: 766-776.

3. Verhaak, RG, Hoadley, KA, Purdom, E, Wang, V, Qi, Y, Wilkerson, MD*, et al.* (2010). Integrated genomic analysis identifies clinically relevant subtypes of glioblastoma characterized by abnormalities in PDGFRA, IDH1, EGFR, and NF1. *Cancer cell* **17**: 98-110.

4. Wang, Q, Hu, B, Hu, X, Kim, H, Squatrito, M, Scarpace, L*, et al.* (2018). Tumor Evolution of Glioma-Intrinsic Gene Expression Subtypes Associates with Immunological Changes in the Microenvironment. *Cancer cell* **33**: 152.

5. Reifenberger, G, Weber, RG, Riehmer, V, Kaulich, K, Willscher, E, Wirth, H*, et al.* (2014). Molecular characterization of long-term survivors of glioblastoma using genome- and transcriptome-wide profiling. *Int J Cancer* **135**: 1822-1831.

6. Reifenberger, G, Wirsching, HG, Knobbe-Thomsen, CB, and Weller, M (2017). Advances in the molecular genetics of gliomas - implications for classification and therapy. *Nat Rev Clin Oncol* **14**: 434-452.

7. Ceccarelli, M, Barthel, FP, Malta, TM, Sabedot, TS, Salama, SR, Murray, BA, *et al.* (2016). Molecular Profiling Reveals Biologically Discrete Subsets and Pathways of Progression in Diffuse Glioma. *Cell* **164**: 550-563.

8. Cancer Genome Atlas Research, N (2008). Comprehensive genomic characterization defines human glioblastoma genes and core pathways. *Nature* **455**: 1061-1068.

9. Ozawa, T, Riester, M, Cheng, YK, Huse, JT, Squatrito, M, Helmy, K*, et al.* (2014). Most human non-GCIMP glioblastoma subtypes evolve from a common proneural-like precursor glioma. *Cancer cell* **26**: 288-300.

10. Cimino, PJ, Kim, Y, Wu, HJ, Alexander, J, Wirsching, HG, Szulzewsky, F*, et al.* (2018). Increased HOXA5 expression provides a selective advantage for gain of whole chromosome 7 in IDH wild-type glioblastoma. *Genes Dev* **32**: 512-523.

11. Goldman, M, Craft, B, Hastie, M, Repečka, K, Kamath, A, McDade, F*, et al.* (2019). The UCSC Xena platform for public and private cancer genomics data visualization and interpretation. *bioRxiv*: 326470.
