# Supplementary material for: Negative allosteric modulators of metabotropic glutamate receptor 3 target the stem-like phenotype of glioblastoma
Source: Mol Ther Oncolytics. 2020 Dec 25;20:166–74. doi: 10.1016/j.omto.2020.12.009 (PMC7851500; doi:10.1016/j.omto.2020.12.009)
Supplement: Document S2. Article plus supplemental information [file mmc2.pdf]

Molecular Therapy
Oncolytics
Original Article

Negative allosteric modulators of metabotropic glutamate receptor 3 target the stem-like phenotype of glioblastoma

Hans-Georg Wirsching,[1,4] Manuela Silginer,[1,4] Elisa Ventura,[1] Will Macnair,[2] Isabel Burghardt,[1] Manfred Claassen,[2] Silvia Gatti,[3] Jürgen Wichmann,[3] Claus Riemer,[3] Hannah Schneider,[1] and Michael Weller[1]

[1]Department of Neurology, University Hospital Zurich and University of Zurich, 8091 Zurich, Switzerland; [2]Institute of Molecular Systems Biology, ETH Zurich, 8093 Zurich, Switzerland; [3]Roche Pharma Research & Early Development, Roche Innovation Center Basel, F. Hoffmann-La Roche Ltd., 4070 Basel, Switzerland

Glioblastoma is an invariably deadly disease. A subpopulation of glioma stem-like cells (GSCs) drives tumor progression and treatment resistance. Two recent studies demonstrated that neurons form oncogenic glutamatergic electrochemical synapses with post-synaptic GSCs. This led us to explore whether glutamate signaling through G protein-coupled metabotropic receptors would also contribute to the malignancy of glioblastoma. We found that glutamate metabotropic receptor (Grm)3 is the predominantly expressed Grm in glioblastoma. Associations of *GRM3* gene expression levels with survival are confined to the proneural gene expression subtype, which is associated with enrichment of GSCs. Using multiplexed single-cell qRT-PCR, GSC marker-based cell sorting, database interrogations, and functional assays in GSCs derived from patients' tumors, we establish Grm3 as a novel marker and potential therapeutic target in GSCs. We confirm that Grm3 inhibits adenylyl cyclase and regulates extracellular signal-regulated kinase. Targeting Grm3 disrupts self-renewal and promotes differentiation of GSCs. Thus, we hypothesize that Grm3 signaling may complement oncogenic functions of glutamatergic ionotropic receptor activity in neuroglial synapses, supporting a link between neuronal activity and the GSC phenotype. The novel class of highly specific Grm3 inhibitors that we characterize herein have been clinically tested as cognitive enhancers in humans with a favorable safety profile.

INTRODUCTION

Glioblastoma is a fatal disease. Despite tremendous progress in the characterization of the molecular landscape of glioblastoma, molecularly targeted therapies have entered clinical practice,[1] and only about 5% of all patients diagnosed with glioblastoma survive for 5 years.[2] Recent single-cell gene expression studies indicate that stem-like gene expression programs in a subset of undifferentiated tumor cells drive the malignant phenotype of gliomas.[3,4] Therefore, therapeutic strategies seeking to specifically target these glioma stem-like cells (GSCs) are considered a promising approach to overcome the malignant phenotype of gliomas.[5] Effective approaches to exploit this vulnerability of gliomas are lacking.

Two recent studies identified oncogenic bona fide glutamatergic synapses between neurons and a subset of glioma cells.[6,7] Overlap of post-synaptic and stem-like gene expression patterns on single-cell RNA sequencing analyses suggests a role of glutamate signaling for the regulation of the GSC phenotype that may be exploited therapeutically.[4,7]

We reasoned that metabotropic glutamate receptors may be candidate mediators of stemness in response to glutamate signaling. G_i protein-coupled glutamate metabotropic receptor (Grm)3 is expressed at high levels by neural stem cells, promoting growth and proliferation.[8] Modulation of intracellular signaling cascades via activation of Grm3 has been implicated in resistance of glioblastoma to chemotherapy by maintaining the GSC phenotype.[9] Notably, Grm3 can be targeted pharmacologically utilizing Grm2/3 negative allosteric modulators, i.e., compounds that exert an activity-dependent decrease in signaling activity in the absence of full receptor blockade. A member of this novel class of drugs demonstrated a favorable safety profile in patients with depressive disorders (ClinicalTrials.gov: NCT01457677).[10]

In summary, this led us to explore Grm as potential therapeutic targets in glioblastomas, seeking to target the GSC phenotype to overcome the malignant phenotype of these tumors.

RESULTS

Grm3 is the predominantly expressed Grm subtype in glioblastoma

As a first step to assess a putative role of Grm in glioblastoma, we queried publically available gene expression and clinical data of The Cancer Genome Atlas (TCGA). Of the eight known Grm subtypes, only *GRM3* is expressed at high levels in glioblastoma (Figure 1A,

Received 19 August 2020; accepted 21 December 2020;
https://doi.org/10.1016/j.omto.2020.12.009.

[4]These authors contributed equally

Correspondence: Hans-Georg Wirsching, PhD, Department of Neurology, University Hospital and University of Zurich, Frauenklinikstrasse 26, 8091 Zurich, Switzerland.
E-mail: hans-georg.wirsching@usz.ch

This is an open access article under the CC BY license (http://creativecommons.org/licenses/by/4.0/).

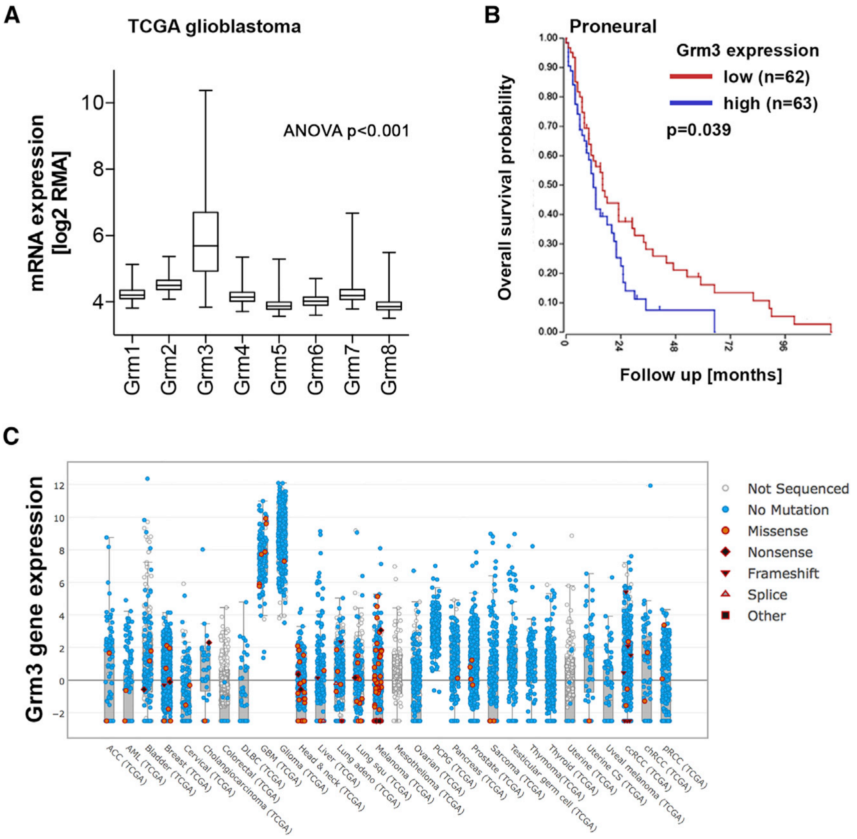

Figure 1. Gene expression of metabotropic glutamate receptors in glioblastoma

(A) *Grm1–Grm8* expression levels in glioblastoma samples (n = 539) from The Cancer Genome Atlas (TCGA) were accessed through the Xena Functional Genomics Explorer: https://xena.ucsc.edu.[11] The box indicates mean and SEM, whiskers represent the range. (B) Kaplan-Meier curve of patients with proneural glioblastoma stratified by *GRM3* gene expression levels (cutoff: median *GRM3* gene expression). (C) Cross-cancer study of *GRM3* mutational status determined by next-generation sequencing and *GRM3* gene expression. Data from TCGA were accessed through the cBIO portal: www.cbioportal.org.[12]

ANOVA, $p < 0.001$). *GRM3* is also the most strongly expressed metabotropic glutamate receptor in the developing and adult non-tumor-bearing brain (Brainspan: www.brainspan.org). A negative association of *GRM3* gene expression levels with overall survival of patients was confined to the proneural glioblastoma gene expression subtype ($p = 0.039$, Figure 1B), which is associated with a more stem-like gene expression phenotype.[13] No association of Grm3 gene expression with survival was apparent in the neural, mesenchymal, or classical subtypes (Figures S1A–S1C). Of note, the neural subtype reflects mostly normal brain expression patterns in samples with low tumor cell content, e.g., in the infiltration zone of tumors.[14,15] The survival association in proneural glioblastoma was independent of the absence or presence of mutations in the genes encoding isocitrate dehydrogenase (*IDH*)-1 or *IDH*-2 (Figures S1D–S1F; Note S1), a molecular marker that defines a clinically and molecularly distinct glioma entity with proneural gene expression. Cross-cancer analyses revealed that gliomas express particularly high levels of Grm3, whereas missense mutations are rare (Figure 1C). Along these lines, single-cell RNA sequencing studies identified the highest *GRM3* gene expression levels in glial cells of the normal mammalian brain[16] and in a subset of tumor cells in human glioblastomas[13] (Single Cell Portal: https://singlecell.broadinstitute.org/single_cell). Of note, *GRM3* is located on chromosome 7, which is commonly amplified in IDH wild-type glioblastoma, yet *GRM3* copy number gains appear not to be associated with gene expression (Note S2; Figures S1G–S1I). Given predominant physiologic expression of Grm3 in the brain, high *GRM3* expression in gliomas may reflect the tissue of origin of these tumors.

Grm3 is co-expressed with GSC markers

Predominant expression of *GRM3* in proneural glioblastoma led us to explore whether *GRM3* was indeed expressed at higher levels by GSCs. We performed quantitative reverse-transcriptase polymerase chain reaction (qRT-PCR) of Grm3 and a panel of neuroglial differentiation markers in 482 single glioblastoma cells derived from six freshly dissociated patient tumors. Table S1 summarizes clinical and molecular characteristics of the donor tumors. We identified positive correlations of *GRM3* gene expression with the GSC markers *OCT4* and *NANOG* whereas expression levels of the differentiation markers *TUBB3* and *GALC* were negatively correlated with *GRM3* (Figure 2A). The overall trend toward a positive correlation of *GRM3* gene expression with GSC markers versus a negative correlation with differentiation markers persisted throughout individual patient samples (Figure S2A). Other genes expressed at higher levels in high versus low *GRM3*-expressing cells include the GSC marker genes *SOX2, SOX4, L1CAM*, and *KLF4*, the DNA repair gene O^6-methylguanine DNA methyltransferase (*MGMT*), and the proneural subtype marker *PDGFA* (Figure S2B).

Several cell surface markers enriched in GSCs have been identified and appear to be associated with distinct glioblastoma subtypes,[18–21] but none of these markers definitely discriminates between GSCs and non-GSCs.[5] We focused on CD133 to explore *GRM3* expression in GSCs versus non-GSCs because the presence of the AC133 epitope of CD133 on the cell surface, but not CD133 gene expression, enriches for GSCs and is associated with the proneural glioblastoma subtype.[20] We first interrogated previously published gene expression data of freshly dissected glioblastoma samples that were sorted for CD133. *GRM3* gene expression was higher in the CD133-positive cell population in seven of eight tumors (Figure 2B), supporting that Grm3 is preferentially expressed in GSCs. Seeking to validate these data, we assessed gene expression levels of both class II metabotropic glutamate receptors, Grm2 and Grm3, in CD133-positive versus CD133-negative cells. In three of five freshly dissected human glioblastomas, gene expression levels of Grm3

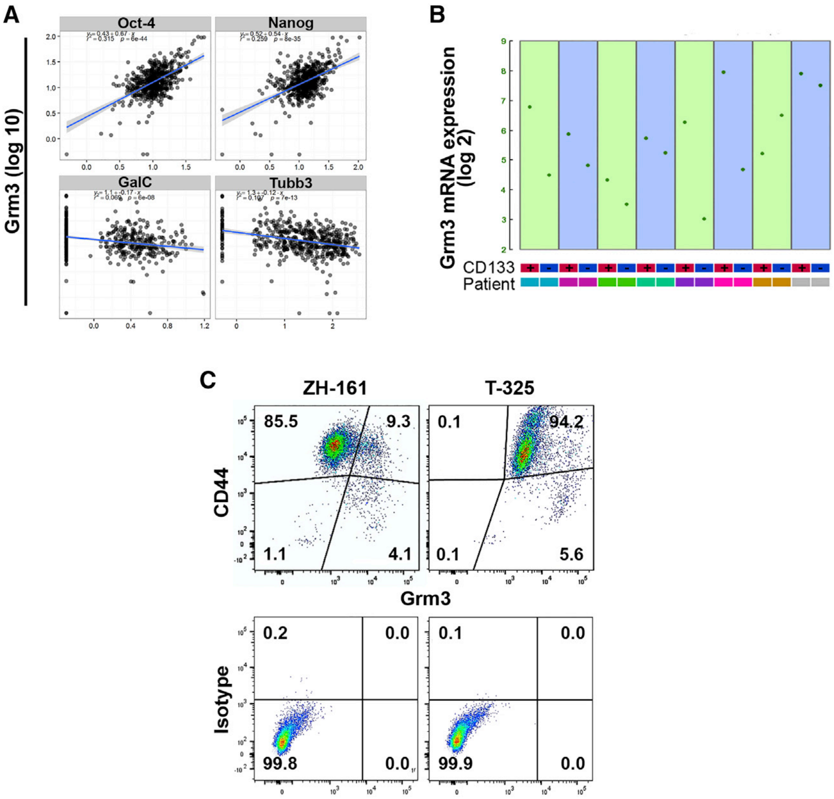

Figure 2. Grm3 expression in glioma-initiating cells

(A) qRT-PCR in single cells (N = 482) derived from freshly dissociated human glioblastomas (N = 6, Table S1). x axis: gene expression of indicated genes. y axis: *GRM3* gene expression. Slopes of blue lines indicate correlations. (B) *GRM3* gene expression sorted for CD133 and analyzed by Affymetrix U133 Plus 2 arrays in a publically available dataset.[17] (C) Flow cytometry of indicated GSCs utilizing fluorescence-labeled antibodies to Grm3 and CD44 or immunoglobulin G (IgG) isotype controls. Numbers in gates indicate percentages.

were higher in the CD133-positive GSC population than in CD133-negative non-GSCs, and Grm3 gene expression was overall higher than that of Grm2 (Figure S2C). These analyses support the heterogeneity of *GRM3* gene expression among glioblastoma cells and support the notion of co-expression of *GRM3* with other stem cell enrichment markers. In glioblastoma cell culture models, Grm2 and Grm3 gene expression levels were also higher in primary cultures maintained under conditions that retain the GSC phenotype, as compared to long-term glioma cells cultured in serum-containing differentiation medium (Figure S2D).

Flow cytometry utilizing the *in vitro* GSC marker CD44[5] confirmed the stem-like phenotype of the majority of cells in the ZH-161 and T-325 GSC models. In ZH-161, Grm3-positive cells constituted a subpopulation of approximately 15% of all cells and these were mostly CD44-positive. In contrast, all T-325 GSCs were positive for Grm3, including the entire CD44-positive population. These differences indicate that low versus high Grm3 mRNA expression in ZH-161 versus T-325 GSCs (Figure 2C) reflects different extents of Grm3-positive cell populations rather than gene expression levels within single cells. In summary, these data indicate that Grm3 is preferentially expressed by GSCs.

Pharmacologic targeting of Grm3 inhibits cyclic adenosine monophosphate and extracellular signal-regulated kinase signaling

Next, we utilized two Grm2/3 negative allosteric modulators, RO4432717 (RO1) and RO0711371 (RO2), to confirm previously reported biological consequences of Grm3 inhibition. The high specificity of these compounds for targeting Grm2/3 at nanomolar concentrations has been determined in a broad Cerep screen of binding affinity to >100 target receptors including other Grm subtypes, identifying half-maximal inhibitory concentration (IC_{50}) values of both compounds above 5 μM for any receptors other than Grm2/3.[22] In order to achieve optimal receptor blockade in the absence of off-target effects, we performed subsequent *in vitro* experiments utilizing RO1 or RO2 at 100 nM. Downstream inhibition of cyclic adenosine monophosphate (cAMP) and extracellular signal-regulated kinase (ERK)/mitogen-activated protein kinase (MAPK) signaling upon targeting Grm3 has been determined previously.[9] Target inhibition in glioma cells was confirmed by a FRET-based cAMP assay where RO1 prevented forskolin-stimulated formation by the Grm2/3 agonist LY-379268 (Figure 3A). Furthermore, RO1 or RO2 expectedly prevented ERK phosphorylation (Figure 3B). In addition, we also considered that class II Grm might modulate G protein-activated inward rectifying potassium channels (GIRKs), but patch clamp recordings in T-325 and T-269 GSCs failed to demonstrate an effect of RO1 or RO2 on GIRK currents (data not shown).

Pharmacological inhibition of Grm3 does not affect chemosensitivity

Chemosensitization of GSCs to temozolomide (TMZ) via downregulation of *MGMT* expression has been reported.[9] The DNA repair protein MGMT is a key mediator of resistance to TMZ in newly diagnosed glioblastoma.[23,24] Therefore, we first assessed growth of two GSC lines with low (S24) versus high (T-325) MGMT protein levels[25] and performed co-treatments with RO1 and TMZ. RO1 inhibited growth of both cell lines at nanomolar concentrations, and TMZ had an additive effect that was more pronounced in S24 (Figure 3C). Similar results were observed upon *GRM3* gene silencing utilizing small interfering RNA (siRNA) (Figure S3A). There was no modulation of MGMT protein levels by RO1 alone or in combination with TMZ, although TMZ expectedly reduced (consumed) MGMT (Figure 3D). In non-GSC lines with established MGMT expression levels and half-maximal effective concentration (EC_{50}) for TMZ,

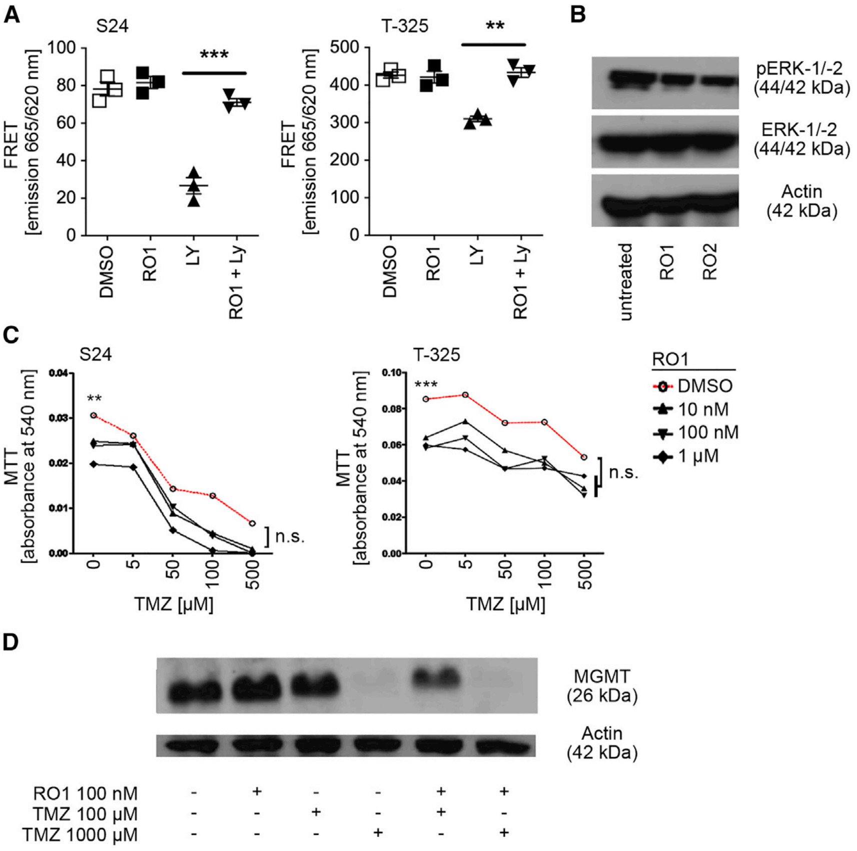

Figure 3. Downstream targets of Grm3 signaling

(A) Fluorescence resonance energy transfer (FRET)-based analysis of forskolin-induced cyclic adenosine monophosphate (cAMP) levels upon treatment with solvent (DMSO), Grm2/3 antagonist (RO1) at 100 nM, Grm2/3 agonist LY-379268 (LY) at 100 nM, or both (**p < 0.01, ***p < 0.001, t test). (B) Immunoblot of indicated proteins. ZH-161 cells were incubated in glutamate- and serum-free medium overnight prior to treatment for 5 min with RO1 or RO2 at 100 nM. (C) Clonogenic survival assay of indicated GSCs co-treated with RO1 or temozolomide (TMZ) or both; 100 cells per well were seeded in 96-well plates in six technical replicates and treated after 24 h. Metabolic activity was assessed utilizing the MTT (3-(4,5-dimethylthiazol-2-yl)-2,5-diphenyltetrazolium bromide) assay on days 7 (S24) or 21 (T-325; **p < 0.01, ***p < 0.001, t test, referring to mono-treatment with RO1 at 100 nM versus DMSO solvent control; n.s., not significant, two-way ANOVA followed by Tukey's post hoc test referring to concentration response of TMZ in RO1 versus DMSO). (D) Immunoblot of MGMT in T-325 cells treated with DMSO, RO1, and TMZ as indicated for 48 h.

inhibition of Grm2/3 by RO1 or RO2 likewise had no effect on the sensitivity to TMZ (Figure S3B), including two cell lines with high MGMT protein levels (LN-18, T98G) and six cell lines without detectable MGMT (U87MG, LN-428, LN-319, LN-229, A172, LN-308).[26] In contrast to GSCs treated with RO1 or RO2, Grm2/3 inhibition in non-GSCs had no effect on clonogenic survival (Figure S3).

Targeting Grm3 induces differentiation and inhibits spherogenicity of GSCs

This led us to evaluate whether growth inhibition was associated with loss of the GSC phenotype, which is characterized by self-renewal and differentiation.[5] Differentiation of GSCs is reflected *in vitro* by the loss of neurosphere formation and adherence of cells to culture dishes. Fetal calf serum (FCS) lowers the threshold of GSCs to adopt this differentiated phenotype. Upon culturing of GSCs for 5 days in the presence of 1% FCS and 100 nM RO1, but not in the presence of FCS without RO1, neurosphere formation of S24 GSCs was almost entirely abrogated in favor of adhesive growth (Figure 4A). We similarly observed attachment of neurospheres and outgrowth of an adhesive cell layer in T-269 cells, although the particularly strong intercellular contacts of T-269 neurospheres were not entirely dissolved (Figure 4A). Next, we assessed the expression of a panel of GSC marker genes in S24 GSCs in response to Grm3 targeting in the absence of FCS. GSC markers were downregulated by either RO1 treatment with 100 nM for 72 h or siRNA-mediated *GRM3* gene silencing (Figure 4B). Treatment with RO1 or RO2 at 100 nM inhibited self-renewal in sphere formation assays of five GSC lines by 12%–54% (mean 30%), including in S24 GSCs by 25% (p = 0.015, RO1) and 17% (p = 0.042, RO2), respectively, and in ZH-161 GSCs by 39% (p = 0.008, RO2) and 31% (p = 0.029, RO2, Figure 5A). Similar effects were observed when spherogenicity was assessed in a limiting dilution assay (Figure 5B) or utilizing a range of RO1 or RO2 concentrations between 10 nM and 1 µM (Figure 5C). Likewise, siRNA-mediated knockdown of Grm3 inhibited sphere formation of S24 GSCs by 64% (p < 0.001) and of ZH-161 GSCs by 43% (p < 0.001, Figure 5D). Dynamic assessments of exhaustion of sphere formation capacity by serial sphere formation assays were not done.

Limited *in vivo* efficacy of pharmacological Grm3 inhibition

Given the similar structure and specificity of RO1 and RO2,[22] and the similar effects of both drugs *in vitro*, we focused our efforts to explore effects of Grm2/3 inhibition *in vivo* on RO1 to limit the number of utilized animals in accordance with the 3R (replacement, reduction, and refinement) principle.[27] The *in vivo* dose of RO1 (30 mg/kg) was selected based on dose-finding experiments conducted by Roche, the provider of RO1, who determined 100 mg/kg as the maximum tolerated dose (data not shown). In the ZH-161 GSC xenograft model, daily treatment with RO1 (30 mg/kg) for 2 weeks inhibited tumor growth by 71% compared to mice treated with a solvent control (N = 3 mice/group, p < 0.05), but not in the T-325 GSC xenograft model (Figure 6A), and no effect of RO1 on apoptosis, invasiveness, or survival of mice bearing either ZH-161 or T-325 glioblastomas was observed (Figures 6B–6D). *In vivo* gene silencing experiments were precluded, because only transient gene silencing could be achieved

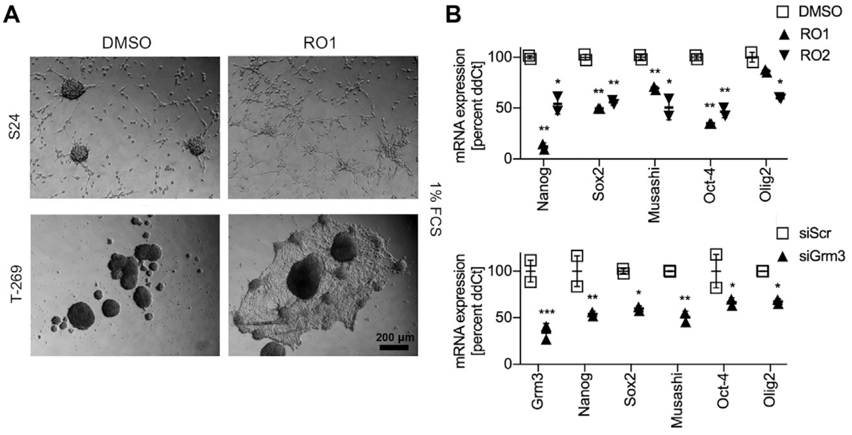

Figure 4. Inhibition of Grm3 signaling induces differentiation of GSCs

(A) Differentiation assay of indicated GSCs treated with DMSO or RO1 at 100 nM. 20,000 cells were seeded in 24-well plates in the presence of 1% FCS for 5 days. (B) mRNA expression levels of indicated genes in S24 GSCs 72 h after treatment with RO1 or RO2 at 100 nM (upper panel), or 72 h after siRNA-mediated *GRM3* gene silencing (lower panel, siGRM3) compared to DMSO or a scrambled control siRNA (siScr), respectively (*p < 0.05, **p < 0.01, ***p < 0.001, two-sided t test corrected for multiple testing utilizing the Holm-Sidak method; error bars indicate SEM).

in vitro (data not shown). In summary, these data suggest that the stem-like phenotype of GSCs can be targeted to inhibit tumor growth utilizing negative allosteric inhibitors of Grm3, although monotherapy approaches targeting Grm3 may not suffice to overcome the malignant phenotype of glioblastoma.

DISCUSSION

Novel treatment approaches against glioblastoma are urgently needed. A key challenge to effectively treating glioblastoma is posed by the extensive cellular heterogeneity of these tumors.[3,13] Cell cycle gene expression is enriched in two stem-like cellular states designated oligodendrocyte progenitor cell-like and neural progenitor cell-like,[3] supporting a concept of cellular hierarchy where tumor progression is driven by undifferentiated GSCs at the apex giving rise to more differentiated bulk tumor cells.[5]

Using database interrogations and gene expression analyses of human single and fluorescence-assisted sorted glioblastoma cells along with functional studies, our experiments indicate that Grm3 is enriched in GSCs and that glutamate signaling through Grm3 may play a pivotal role for the maintenance of the GSC phenotype. In contrast, non-GSCs were unaffected by Grm3 inhibition. Pharmacologic inhibition of Grm3 signaling utilizing two members of a novel class of negative allosteric modulators of Grm3 signaling inhibited self-renewal, GSC marker expression, and *in vivo* tumor growth in one of two GSC xenograft models. Notably, survival was not prolonged by this monotherapy approach. The observed growth inhibitory effect in one model and previous pharmacokinetic studies suggest that insufficient drug delivery was unlikely the reason for this discrepancy. One possible explanation may be that GSCs tend to adopt proneural features upon *in vitro* culturing,[21] while the utilized models expressed CD44, a marker of the mesenchymal subtype, which appeared to be less dependent on Grm3 in our database analyses. The lack of an effect of Grm3 inhibition on tumor invasion and failure to induce apoptosis suggest that mere targeting of the GSC state may not suffice to improve outcome and warrants combination treatment approaches to exploit potential synergy with cytotoxic treatment approaches, albeit we observed no synergy with TMZ *in vitro*. Considering that GSCs give rise to faster cycling bulk tumor cells, we further speculate that the observed moderate *in vitro* inhibition of GSC self-renewal by Grm3 inhibition may still exert relevant long-term effects on overall tumor growth.

A previous study investigating a role of Grm3 for GSCs suggested sensitization to TMZ upon Grm3 inhibition through transcriptional suppression of *MGMT*,[9] but we observed no more than additive effects of Grm3 inhibition and TMZ and no modulation of MGMT expression by RO1. Radiotherapy schedules have been optimized by mathematical modeling to specifically target vulnerable states of GSCs.[28] Targeting Grm3 may promote cycling of GSCs toward more vulnerable cellular states, warranting further mathematical exploration and pre-clinical validation of synergy of combined radiotherapy and Grm3 inhibition.

Our findings expand on two recent reports of glutamate signaling via bona fide synapses between neurons and post-synaptic, presumably GSC-like, cells, both of which focused on ionotropic glutamate receptor signaling.[6,7] Beyond synaptic transmission, glutamate is also released excessively into the tumor microenvironment by glioma cells,[29,30] supporting the progression of gliomas via autocrine and paracrine activation of ionotropic α-amino-3-hydroxy-5-methyl-4-isoxazolepropionic acid (AMPA)-type ionotropic glutamate receptors[30] and inducing neuronal cell death through hyperactivation of inward calcium currents via *N*-methyl-D-aspartate (NMDA)-type glutamate receptors.[31] By enhancing the excitability of neurons in the tumor microenvironment and promoting epilepsy,[29] glutamate signaling may moreover promote synaptic transmission to activate GSCs. Whether Grm3 is activated primarily in an autocrine and paracrine manner, synaptically, or both remains to be determined.

Notably, glutamate signaling in glioblastoma can be targeted pharmacologically at multiple levels utilizing drugs that are well established in clinical practice for other purposes. The anti-epileptic drug gabapentin can lower the interconversion of α-ketoglutarate to glutamate,[32] and the anti-inflammatory drug sulfasalazine inhibits the cystine/glutamate antiporter system x_c,[29] with both resulting in decreased glutamate secretion into the tumor microenvironment to inhibit neuronal excitation and thus presumably reduce the activity of neuron-glioma synapses. The anti-dementia drug memantine may

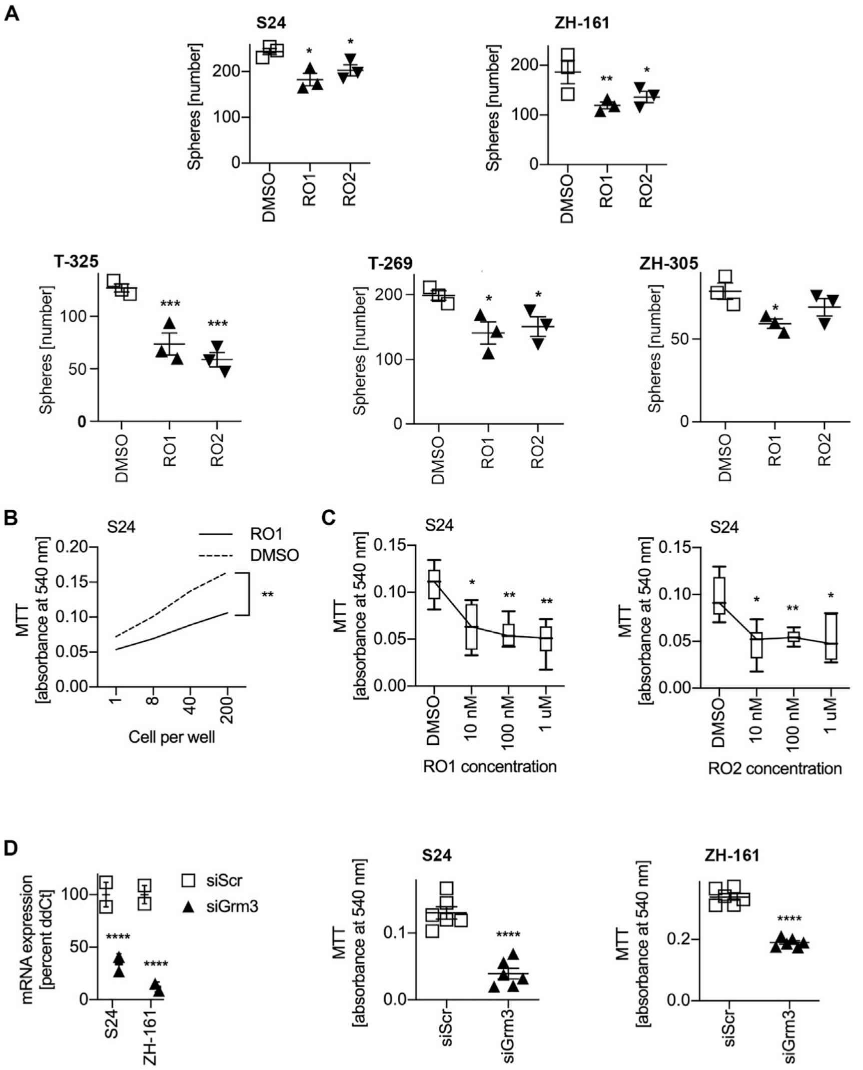

Figure 5. Inhibition of Grm3 signaling impairs spherogenicity of GSCs

(A) Spherogenicity assay of indicated GSC lines. Upper panel: 500 cells were seeded in triplicates as a single-cell suspension in 2 mL of neurobasal medium and treated with RO1 or RO2 at 100 nM after 24 h. Sphere numbers were counted manually after 21 days (*p < 0.05, **p < 0.01, ***p < 0.001, two-sided non-parametric t test followed by Holm-Sidak correction for multiple testing; error bars indicate SEM). (B) Limiting dilution assay of S24 cells seeded in triplicate in neurobasal medium and treated with 100 nM RO1 24 h later. Analysis by MTT was done after 10 days (**p < 0.01, two-way ANOVA followed by Tukey's test). (C) Concentration response to RO1 (left panel) and RO2 (right panel). S24 cells were seeded at 100 cells per well in 96-well plates and treated with indicated drug concentrations. Analysis by MTT was done after 10 days (*p < 0.05, **p < 0.01, multiple t tests utilizing DMSO as the comparator were done and corrected utilizing the Holm-Sidak method). (D) Confirmation of siRNA-mediated Grm3 gene silencing (left panel). qRT-PCR utilizing the ΔΔCT method, and housekeeping gene normalization was done 72 h after transfection with siRNA directed against Grm3 mRNA (siGrm3) or a scrambled control siRNA (siScr). Grm3 expression is depicted as mean percentage of siScr (****p < 0.0001, two-sided t test; error bars indicate SEM). Middle and right panels: 72 h after siRNA-mediated *GRM3* gene silencing, 100 cells per well were seeded in 96-well plates in six technical replicates and metabolic activity was assessed utilizing the MTT assay on day 7 (****p < 0.001, t test corrected for multiple testing utilizing the Holm-Sidak method; error bars indicate SEM).

have neuroprotective effects to counteract against excessive NMDA-type glutamate receptor activation,[31] and the anti-epileptic AMPA-type glutamate receptor inhibitor perampanel may directly counteract oncogenic synaptic signaling between neurons and glioma cells.[6,7] Therefore, negative allosteric modulators of Grm3 may also be explored in combination with other anti-glutamatergic drugs to counteract the oncogenic effects of glutamate at multiple levels.

Of note, despite the strong rationale for glutamate-targeted therapy approaches of gliomas, the central clinical study registration portal of the US ClinicalTrials.gov lists only two such studies as of November 22, 2020. One ongoing study in glioblastoma patients explores the efficacy of the anti-alcoholic drug disulfiram, which is thought to inhibit glutamate uptake in the brain, with the primary endpoint being overall survival (ClinicalTrials.gov: NCT02715609). The other glutamate-targeted study listed is in the planning phase and will include any glioma patients with drug-resistant epilepsy for treatment with perampanel or a standard anti-epileptic drug regimen (ClinicalTrials.gov: NCT03636958). Although the primary endpoint of the latter study is reduction in seizure frequency, survival will also be recorded and likely be reported post hoc. A third study explored the feasibility of a specific diet to reduce intratumoral glutamate levels as determined by magnetic resonance spectroscopy, with results being awaited in due time (ClinicalTrials.gov: NCT02286167). Thus, there is an unmet need of clinical studies exploring the efficacy of glutamate targeting in glioma patients.

Supporting the feasibility of future exploration of Grm3 inhibition in glioma patients, a similar negative allosteric modulator of Grm2/3 (decoglurant) was well tolerated by patients treated in a phase II clinical trial of major depressive disorder (ClinicalTrials.gov: NCT01457677). Along the same lines, treatment applications of Grm2/3 negative allosteric modulators for other psychiatric disorders have been suggested based on promising preclinical and preliminary clinical data, including cognitive enhancement, psychotic disorders, and sleep-wake disorders.[33]

The presence of a negative correlation of *GRM3* gene expression with survival in glioblastomas with proneural gene expression, but not in patients with classical and mesenchymal glioblastoma, is of note,

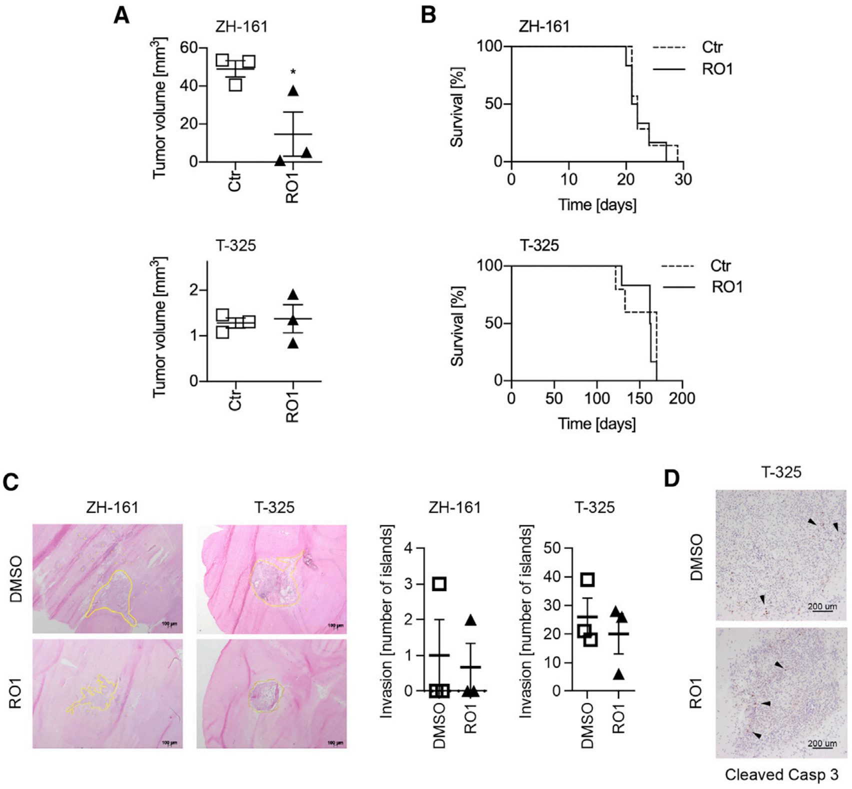

Figure 6. Limited *in vivo* effects of RO1

Tumors were generated by orthotopic injection of 100,000 ZH-161 or T-325 cells in nude mice. Beginning on day 14 after implantation, mice were treated daily for 7 days by oral gavage with RO1 (30 mg/kg in 0.3% Tween 20 in saline) or solvent control. Three animals per group were euthanized on day 21 after tumor cell implantation for tissue analyses. (A) Tumor volumes were determined by area measurement on at least five hematoxylin and eosin (H&E)-stained slides per tumor utilizing ImageJ followed by calculation per stack height. (B) Survival was defined as the time from tumor cell implantation to the development of neurological symptoms or loss of at least 15% weight (N = 5–7 mice per group, log rank test not significant). (C) Tumor invasion was determined by counting of tumor cell islands, defined as at least 50 cells at a distance of at least two cell layers from the tumor bulk (yellow lines). (D) Apoptosis was determined by cleaved caspase-3 staining (arrowheads indicate groups of caspase-3-positive cells).

because on the single-cell level, proneural gene expression is associated with the expression of stemness-related genes.[13] Although glioblastoma gene expression subtypes are associated with distinctive genomic features,[34,35] these oncogenic differences may merely result in a different cell type composition. Whether targeting Grm3 should be confined to proneural glioblastoma remains elusive, because Grm3-positive GSCs may as well reside in other subtype glioblastomas at lower frequencies and may likewise drive the malignant phenotype of these tumors,[21] and associations of the expression of single genes with survival need to be interpreted with caution.

In summary, Grm3 may be a novel, pharmacologically accessible target in glioblastomas, although future studies will be required to decipher the precise molecular mechanism of potential anti-glioma activity of Grm3 inhibitors. To date, drugs to specifically target the GSC phenotype are lacking. Clinical exploration of Grm3 targeting in glioblastoma patients is warranted.

MATERIALS AND METHODS

Extended Supplemental materials and methods are available online.

Database interrogations

Publically available microarray and clinical data on glioma patients,[36–38] and genomic data for cross-cancer analyses, were acquired from TCGA. Data were accessed on October 31, 2019 and analyzed utilizing the web tools cBIO portal (www.cbioportal.org)[12] and Xena (https://xena.ucsc.edu)[11], or the R2 genomics analysis and visualization platform (https://hgserver1.amc.nl/cgi-bin/r2/main.cgi).[40] Glioblastoma gene expression subtypes were defined according to Verhaak et al.[34]

Glioblastoma tissue samples

Glioblastoma samples were collected from patients who were treated at the Department of Neurosurgery, University Hospital Zurich, Zurich, Switzerland. Written informed consent was obtained from all individuals and tissue collection was approved by the local ethics committee.

Cells and reagents

The GSC lines ZH-161, ZH-305, S24, T-325, and T-269 were established from freshly resected tumors and cultured as sphere cultures under stem cell conditions. All other cell lines were cultured under standard adherent conditions. Stem cell conditions for culturing GSCs were neurobasal medium supplemented with B-27 (20 µL/mL; Invitrogen), GlutaMAX (10 µL/mL; Invitrogen), fibroblast growth factor-2, epidermal growth factor (20 ng/mL each; PeproTech), and heparin (32 IU/mL; Ratiopharm). Growth factors were replenished twice weekly. The non-GSC LN-18, LN-229, LN-308, LN-319, and LN-428 glioma cell lines were kindly provided by N. de Tribolet, and T98G, U87MG, and A172 glioma cell lines were purchased from the American Type Culture Collection. Non-GSC cell lines were cultured in Dulbecco's modified Eagle's medium (DMEM) supplemented with 10% FCS. Dimethyl sulfoxide was utilized as a solvent for TMZ (Merck) and Grm2/3 negative allosteric modulators RO1 and RO2 at final concentrations of 0.2%. Grm2/3 negative allosteric modulators RO1 and RO2 were provided by F. Hoffmann-La Roche.

Single-cell qRT-PCR

Glioblastoma tissues were obtained from the operating room and immediately dissociated using a papain-based dissociation system (Worthington Biochemical). Leukocytes were depleted using

anti-human CD45 MicroBeads (Miltenyi Biotec). The C1 single-cell auto prep instrument (Fluidigm) was used for capturing single cells. Pre-amplified cDNA was utilized for qPCR with the Biomark HD system with IFC Controller HX (Fluidigm) and 2× SsoFAST EvaGreen supermix with low ROX (Bio-Rad).

CD133 magnetic-activated cell sorting and qRT-PCR

Separation of freshly dissociated tumor specimens into CD133-positive and CD133-negative cell fractions was performed using MicroBeads conjugated to the mouse anti-human CD133/1 epitope antibody (clone AC133, Miltenyi Biotec). Depletion of CD45-positive cells was followed by separation of CD133-positive and CD133-negative cell populations utilizing magnetic-activated cell sorting (MACS) LS columns (Miltenyi Biotec). All differential mRNA expression data were obtained from cells lysed immediately following magnetic sorting.

cAMP assay

A cell-based competitive immunosorbent assay kit to detect cAMP was utilized (cisbio). Adenyl cyclase stimulation was done with forskolin at 10 μM with or without the Grm2/3 agonist LY-379268 at 100 nM or RO1 or both. Fluorescence resonance energy transfer (FRET) between XL665-labeled cAMP and a cryptate-labeled monoclonal anti-cAMP antibody was measured utilizing a Tecan Infinite plate reader (Tecan).

siRNA-mediated gene silencing

For transient transfections, glioma cells were seeded in six-well plates and transfected with 100 nM specific or scrambled control siRNA by electroporation using the Neon transfection system (Invitrogen). Pools of control and *GRM3* siRNA oligonucleotides were purchased from Thermo Scientific using siGENOME SMARTpool.

Statistical analysis

For column statistics of *in vitro* experiments, an unpaired t test or one-way ANOVA was performed, followed by correction for multiple testing utilizing the Holm-Sidak method or Tukey's post hoc test, as indicated. The *in vitro* experiments reported herein were performed at least two times in triplicate with similar results and analyzed using Prism 8 (GraphPad). The log rank test was applied for survival statistics. Correlations of gene expression in single cells were assessed by an unpaired t test and Bonferroni correction for multiple testing in the statistical environment R (v3.3.2) utilizing the ggplot2 package for graphical display. Statistics for database analyses were performed utilizing the respective web tools through which data were accessed and visualized. Copy number alterations annotated by Xena were calculated by GISTIC2.0 with estimated values −2, −1, 0, 1, and 2 representing homozygous deletion, single copy deletion, diploidy, low-level copy number amplification, or copy number gain, respectively.[39] DNA methylation values annotated by Xena are described as beta values derived at the Johns Hopkins University and University of Southern California TCGA genome characterization center as continuous variables between 0 and 1, representing the ratio of the intensity of the methylated bead type to the combined locus intensity. Throughout the manuscript, p values <0.05 were considered statistically significant.

SUPPLEMENTAL INFORMATION

Supplemental Information can be found online at https://doi.org/10.1016/j.omto.2020.12.009.

ACKNOWLEDGMENTS

The study was supported by a grant from F. Hoffmann-La Roche (to M.W.), the Swiss Life Foundation (to H.S.), and the Betty and David Koetser Foundation (to H.-G.W.). The authors would also like to thank J. Friesen for excellent technical assistance, C. Beisel and K. Eschbach (University of Basel) for assistance with single-cell qRT-PCR analyses, and Dr. F. Knoflach (Roche) for the conduct of the GIRK patch clamp recordings in GSCs with Grm2/3 negative allosteric modulators.

AUTHOR CONTRIBUTIONS

H.-G.W. and M.W. designed the study and interpreted the data; E.V. and I.B. performed single-cell qRT-PCR; W.M., M.C., H.-G.W., and M.S. analyzed the data; H.-G.W. and M.S. created the figures; H.-G.W., M.S., and H.S. collected data; M.W. and H.-G.W. drafted the manuscript; all authors contributed to the writing and revision of the manuscript and approved the final version.

DECLARATIONS OF INTEREST

H.-G.W. and M.W. received compensation for advisory boards of F. Hoffmann-La Roche. M.W. received research funding from F. Hoffmann-La Roche. S.G., J.W., and C.R. were employees of F. Hoffmann-La Roche during the conduct of this study.

REFERENCES

1. Weller, M., van den Bent, M., Tonn, J.C., Stupp, R., Preusser, M., Cohen-Jonathan-Moyal, E., Henriksson, R., Le Rhun, E., Balana, C., Chinot, O., et al.; European Association for Neuro-Oncology (EANO) Task Force on Gliomas (2017). European Association for Neuro-Oncology (EANO) guideline on the diagnosis and treatment of adult astrocytic and oligodendroglial gliomas. Lancet Oncol. *18*, e315–e329.
2. Ostrom, Q.T., Gittleman, H., Fulop, J., Liu, M., Blanda, R., Kromer, C., Wolinsky, Y., Kruchko, C., and Barnholtz-Sloan, J.S. (2015). CBTRUS statistical report: primary brain and central nervous system tumors diagnosed in the United States in 2008–2012. Neuro-oncol. *17* (*Suppl 4*), iv1–iv62.
3. Neftel, C., Laffy, J., Filbin, M.G., Hara, T., Shore, M.E., Rahme, G.J., Richman, A.R., Silverbush, D., Shaw, M.L., Hebert, C.M., et al. (2019). An integrative model of cellular states, plasticity, and genetics for glioblastoma. Cell *178*, 835–849.e21.
4. Filbin, M.G., Tirosh, I., Hovestadt, V., Shaw, M.L., Escalante, L.E., Mathewson, N.D., Neftel, C., Frank, N., Pelton, K., Hebert, C.M., et al. (2018). Developmental and oncogenic programs in H3K27M gliomas dissected by single-cell RNA-seq. Science *360*, 331–335.
5. Lathia, J.D., Mack, S.C., Mulkearns-Hubert, E.E., Valentim, C.L., and Rich, J.N. (2015). Cancer stem cells in glioblastoma. Genes Dev. *29*, 1203–1217.
6. Venkataramani, V., Tanev, D.I., Strahle, C., Studier-Fischer, A., Fankhauser, L., Kessler, T., Körber, C., Kardorff, M., Ratliff, M., Xie, R., et al. (2019). Glutamatergic synaptic input to glioma cells drives brain tumour progression. Nature *573*, 532–538.
7. Venkatesh, H.S., Morishita, W., Geraghty, A.C., Silverbush, D., Gillespie, S.M., Arzt, M., Tam, L.T., Espenel, C., Ponnuswami, A., Ni, L., et al. (2019). Electrical and synaptic integration of glioma into neural circuits. Nature *573*, 539–545.

8. Di Giorgi-Gerevini, V., Melchiorri, D., Battaglia, G., Ricci-Vitiani, L., Ciceroni, C., Busceti, C.L., Biagioni, F., Iacovelli, L., Canudas, A.M., Parati, E., et al. (2005). Endogenous activation of metabotropic glutamate receptors supports the proliferation and survival of neural progenitor cells. Cell Death Differ. *12*, 1124–1133.

9. Ciceroni, C., Bonelli, M., Mastrantoni, E., Niccolini, C., Laurenza, M., Larocca, L.M., Pallini, R., Traficante, A., Spinsanti, P., Ricci-Vitiani, L., et al. (2013). Type-3 metabotropic glutamate receptors regulate chemoresistance in glioma stem cells, and their levels are inversely related to survival in patients with malignant gliomas. Cell Death Differ. *20*, 396–407.

10. Umbricht, D., Niggli, M., Sanwald-Ducray, P., Deptula, D., Moore, R., Grünbauer, W., Boak, L., and Fontoura, P. (2020). Randomized, double-blind, placebo-controlled trial of the mGlu2/3 negative allosteric modulator decoglurant in partially refractory major depressive disorder. J. Clin. Psychiatry *81*, 18m12470.

11. Goldman, M., Craft, B., Hastie, M., Repečka, K., Kamath, A., McDade, F., Rogers, D., Brooks, A.N., Zhu, J., and Haussler, D. (2019). The UCSC Xena platform for public and private cancer genomics data visualization and interpretation. bioRxiv. https://doi.org/10.1101/326470.

12. Cerami, E., Gao, J., Dogrusoz, U., Gross, B.E., Sumer, S.O., Aksoy, B.A., Jacobsen, A., Byrne, C.J., Heuer, M.L., Larsson, E., et al. (2012). The cBio cancer genomics portal: an open platform for exploring multidimensional cancer genomics data. Cancer Discov. *2*, 401–404.

13. Patel, A.P., Tirosh, I., Trombetta, J.J., Shalek, A.K., Gillespie, S.M., Wakimoto, H., Cahill, D.P., Nahed, B.V., Curry, W.T., Martuza, R.L., et al. (2014). Single-cell RNA-seq highlights intratumoral heterogeneity in primary glioblastoma. Science *344*, 1396–1401.

14. Wang, Q., Hu, B., Hu, X., Kim, H., Squatrito, M., Scarpace, L., deCarvalho, A.C., Lyu, S., Li, P., Li, Y., et al. (2018). Tumor evolution of glioma-intrinsic gene expression subtypes associates with immunological changes in the microenvironment. Cancer Cell *33*, 152.

15. Reifenberger, G., Weber, R.G., Riehmer, V., Kaulich, K., Willscher, E., Wirth, H., Gietzelt, J., Hentschel, B., Westphal, M., Simon, M., et al.; German Glioma Network (2014). Molecular characterization of long-term survivors of glioblastoma using genome- and transcriptome-wide profiling. Int. J. Cancer *135*, 1822–1831.

16. Habib, N., Li, Y., Heidenreich, M., Swiech, L., Avraham-Davidi, I., Trombetta, J.J., Hession, C., Zhang, F., and Regev, A. (2016). Div-Seq: single-nucleus RNA-seq reveals dynamics of rare adult newborn neurons. Science *353*, 925–928.

17. Garcia, J.L., Perez-Caro, M., Gomez-Moreta, J.A., Gonzalez, F., Ortiz, J., Blanco, O., Sancho, M., Hernandez-Rivas, J.M., Gonzalez-Sarmiento, R., and Sanchez-Martin, M. (2010). Molecular analysis of ex-vivo $CD133^+$ GBM cells revealed a common invasive and angiogenic profile but different proliferative signatures among high grade gliomas. BMC Cancer *10*, 454.

18. Son, M.J., Woolard, K., Nam, D.H., Lee, J., and Fine, H.A. (2009). SSEA-1 is an enrichment marker for tumor-initiating cells in human glioblastoma. Cell Stem Cell *4*, 440–452.

19. Singh, S.K., Hawkins, C., Clarke, I.D., Squire, J.A., Bayani, J., Hide, T., Henkelman, R.M., Cusimano, M.D., and Dirks, P.B. (2004). Identification of human brain tumour initiating cells. Nature *432*, 396–401.

20. Yan, X., Ma, L., Yi, D., Yoon, J.G., Diercks, A., Foltz, G., Price, N.D., Hood, L.E., and Tian, Q. (2011). A CD133-related gene expression signature identifies an aggressive glioblastoma subtype with excessive mutations. Proc. Natl. Acad. Sci. USA *108*, 1591–1596.

21. Bhat, K.P.L., Balasubramaniyan, V., Vaillant, B., Ezhilarasan, R., Hummelink, K., Hollingsworth, F., Wani, K., Heathcock, L., James, J.D., Goodman, L.D., et al. (2013). Mesenchymal differentiation mediated by NF-κB promotes radiation resistance in glioblastoma. Cancer Cell *24*, 331–346.

22. Woltering, T.J., Wichmann, J., Goetschi, E., Knoflach, F., Ballard, T.M., Huwyler, J., and Gatti, S. (2010). Synthesis and characterization of 1,3-dihydro-benzo[*b*][1,4]diazepin-2-one derivatives: Part 4. In vivo active potent and selective non-competitive metabotropic glutamate receptor 2/3 antagonists. Bioorg. Med. Chem. Lett. *20*, 6969–6974.

23. Weller, M., Stupp, R., Reifenberger, G., Brandes, A.A., van den Bent, M.J., Wick, W., and Hegi, M.E. (2010). *MGMT* promoter methylation in malignant gliomas: ready for personalized medicine? Nat. Rev. Neurol. *6*, 39–51.

24. Hegi, M.E., Diserens, A.C., Gorlia, T., Hamou, M.F., de Tribolet, N., Weller, M., Kros, J.M., Hainfellner, J.A., Mason, W., Mariani, L., et al. (2005). *MGMT* gene silencing and benefit from temozolomide in glioblastoma. N. Engl. J. Med. *352*, 997–1003.

25. Happold, C., Stojcheva, N., Silginer, M., Weiss, T., Roth, P., Reifenberger, G., and Weller, M. (2018). Transcriptional control of O^6-methylguanine DNA methyltransferase expression and temozolomide resistance in glioblastoma. J. Neurochem. *144*, 780–790.

26. Hermisson, M., Klumpp, A., Wick, W., Wischhusen, J., Nagel, G., Roos, W., Kaina, B., and Weller, M. (2006). O^6-methylguanine DNA methyltransferase and p53 status predict temozolomide sensitivity in human malignant glioma cells. J. Neurochem. *96*, 766–776.

27. Törnqvist, E., Annas, A., Granath, B., Jalkesten, E., Cotgreave, I., and Öberg, M. (2014). Strategic focus on 3R principles reveals major reductions in the use of animals in pharmaceutical toxicity testing. PLoS ONE *9*, e101638.

28. Leder, K., Pitter, K., LaPlant, Q., Hambardzumyan, D., Ross, B.D., Chan, T.A., Holland, E.C., and Michor, F. (2014). Mathematical modeling of PDGF-driven glioblastoma reveals optimized radiation dosing schedules. Cell *156*, 603–616.

29. Buckingham, S.C., Campbell, S.L., Haas, B.R., Montana, V., Robel, S., Ogunrinu, T., and Sontheimer, H. (2011). Glutamate release by primary brain tumors induces epileptic activity. Nat. Med. *17*, 1269–1274.

30. Lyons, S.A., Chung, W.J., Weaver, A.K., Ogunrinu, T., and Sontheimer, H. (2007). Autocrine glutamate signaling promotes glioma cell invasion. Cancer Res. *67*, 9463–9471.

31. Takano, T., Lin, J.H., Arcuino, G., Gao, Q., Yang, J., and Nedergaard, M. (2001). Glutamate release promotes growth of malignant gliomas. Nat. Med. *7*, 1010–1015.

32. Tönjes, M., Barbus, S., Park, Y.J., Wang, W., Schlotter, M., Lindroth, A.M., Pleier, S.V., Bai, A.H.C., Karra, D., Piro, R.M., et al. (2013). BCAT1 promotes cell proliferation through amino acid catabolism in gliomas carrying wild-type IDH1. Nat. Med. *19*, 901–908.

33. Celanire, S., Sebhat, I., Wichmann, J., Mayer, S., Schann, S., and Gatti, S. (2015). Novel metabotropic glutamate receptor 2/3 antagonists and their therapeutic applications: a patent review (2005–present). Expert Opin. Ther. Pat. *25*, 69–90.

34. Verhaak, R.G., Hoadley, K.A., Purdom, E., Wang, V., Qi, Y., Wilkerson, M.D., Miller, C.R., Ding, L., Golub, T., Mesirov, J.P., et al.; Cancer Genome Atlas Research Network (2010). Integrated genomic analysis identifies clinically relevant subtypes of glioblastoma characterized by abnormalities in *PDGFRA*, *IDH1*, *EGFR*, and *NF1*. Cancer Cell *17*, 98–110.

35. Ozawa, T., Riester, M., Cheng, Y.K., Huse, J.T., Squatrito, M., Helmy, K., Charles, N., Michor, F., and Holland, E.C. (2014). Most human non-GCIMP glioblastoma subtypes evolve from a common proneural-like precursor glioma. Cancer Cell *26*, 288–300.

36. Brennan, C.W., Verhaak, R.G., McKenna, A., Campos, B., Noushmehr, H., Salama, S.R., Zheng, S., Chakravarty, D., Sanborn, J.Z., Berman, S.H., et al.; TCGA Research Network (2013). The somatic genomic landscape of glioblastoma. Cell *155*, 462–477.

37. Cancer Genome Atlas Research Network (2008). Comprehensive genomic characterization defines human glioblastoma genes and core pathways. Nature *455*, 1061–1068.

38. Ceccarelli, M., Barthel, F.P., Malta, T.M., Sabedot, T.S., Salama, S.R., Murray, B.A., Morozova, O., Newton, Y., Radenbaugh, A., Pagnotta, S.M., et al.; TCGA Research Network (2016). Molecular profiling reveals biologically discrete subsets and pathways of progression in diffuse glioma. Cell *164*, 550–563.

39. Mermel, C.H., Schumacher, S.E., Hill, B., Meyerson, M.L., Beroukhim, R., and Getz, G. (2011). GISTIC2.0 facilitates sensitive and confident localization of the targets of focal somatic copy-number alteration in human cancers. Genome Biol. *12*, R41.

40. Koster, J., Molenaar, J.J., and Versteeg, R. (2015). R2: accessible web-based genomics analysis and visualization platform for biomedical researchers. Cancer Res. *75* (*Suppl 1*), A2-45.

OMTO, Volume *20*

Supplemental Information

Negative allosteric modulators of metabotropic glutamate receptor 3 target the stem-like phenotype of glioblastoma

Hans-Georg Wirsching, Manuela Silginer, Elisa Ventura, Will Macnair, Isabel Burghardt, Manfred Claassen, Silvia Gatti, Jürgen Wichmann, Claus Riemer, Hannah Schneider, and Michael Weller

SUPPLEMENTARY MATERIAL

Supplementary Methods

Single-cell RT-PCR

Glioblastoma tissues were obtained from the operating room and immediately dissociated using a papain-based dissociation system (Worthington). Leukocytes were depleted using anti-human CD45 microbeads (Miltenyi Biotec). Cells were magnetically labeled with microbeads labeled with anti-human CD45 antibody for 15 minutes at +4°C. CD45+ and CD45- cells were separated using MACS LD columns (Miltenyi Biotec). Cells were captured on a C1 Single-Preamp IFC (10-17 µm) using the C1 Single-Cell Autoprep instrument (Fluidigm). The C1 Single-Preamp IFC was imaged under an inverted microscope to identify and exclude empty sites or sites with multiple cells or debris. After the generation of preamplified cDNA using the Single Cells-to-CT kit (Life Technologies), pooled primers and Fluidigm STA reagents, qPCR was performed by using the BioMark HD system with IFC Controller HX (Fluidigm) and 2X ssoFAST EvaGreen Supermix with Low Rox (Bio-Rad). Data were collected using the Fludigim Data Collection software. Quality controls included cDNAs derived from a glioblastoma tissue before and after CD45+ depletion and the qPCR Human Reference cDNA, random-primer (Clontech). All primers were tested for amplification efficiency of at least 90% by generating standard curves for each gene. Data was processed via removal of cells with low overall expression values, identification of limits of detection for each analyzed gene and cell-to-cell median normalization.[1]

The following primers were utilized:

mGlu2 fwd 5'-CACGGCAGTGTGTACCTTACG -3’

mGlu2 rv: 5'- GATGCGTGCAATGCGGTTG -3'

mGlu3 fwd: 5'- AGCAATCACTGGAGTTTGTCAG -3'

mGlu3 rv: 5'- GCAATGAGAAGTGGGATGTTTTC -3'

Oct-4 fwd: 5'- CGAGAAGGATGTGGTCCGAG -3'

Oct-4 rv: 5'- TGTGCATAGTCGCTGCTTGA -3'

Nanog fwd: 5'- GAAATACCTCAGCCTCCAGC -3'

Nanog rv: 5'-GCGTCACACCATTGCTATTC -3'

Musashi fwd: 5'- CCAATGGGTACCACTGAAGC -3'

Musashi rv: 5'- ACTCGTGGTCCTCAGTCAGC -3'

Olig2 fwd: 5'- AGCTCCTCAAATCGCATCC -3'

Olig2 rv: 5'- ATAGTCGTCGCAGCTTTCG -3'

GalC fwd: 5'- GGATCTTACAGGGTTACAGGTGA -3'

GalC rv: 5'- TGCTGTAACTTCAACACGTCCT -3'

Tubb3 fwd: 5'- TCTTCTCACAAGTACGTGCCTC -3'

Tubb3 rv: 5'- GTGTAGTGACCCTTGGCCC -3'

Map2 fwd: 5'- TGCCTCAGAACAGACTGTCAC -3'

Map2 rv: 5'- GGCTCTTGGTTACTCCGTCA -3'

SOX2 fwd: 5'- CACACTGCCCCTCTCAC -3'

SOX2 rv: 5'- TCCATGCTGTTTCTTACTCTC -3'

SOX4 fwd: 5'- GGTCTCTAGTTCTTGCACGCT -3’

SOX4 rv: 5'- CTGCAAGAAGGGAGCTGGTAA -3'

KLF4 fwd: 5’- CTCATGCCACCCGGTTCC -3’

KLF4 rv: 5’- TTTCTCACCTGTGTGGGTTCG -3’

PDGFA fwd: 5’- CGTAGGGAGTGAGGATTCTTTGG -3’

PDGFA rv: 5’- AATGACCGTCCTGGTCTTGC -3’

L1CAM fwd: 5’- CCCCAGGTCACTATCGGCTA -3’

L1CAM rv: 5’- GACTGCCCTCCCTCCAGTA -3’

MGMT fwd: 5’- GTCGTTCACCAGACAGGTGTT -3’

MGMT rv: 5’- ACAGGATTGCCTCTCATTGCTC -3’.
CD133 fwd: 5'- TGGATGCAGAACTTGACAACGT -3'
CD133 rv: 5'-ATACCTGCTACGACAGTCGTGGT-3'
Arf1 fwd: 5’-GACCACGATCCTCTACAAGC -3’
Arf1 rv: 5’- TCCCACACAGTGAAGCTGATG -3’.

CD133 magnetic-activated cell sorting and RT-PCR

Dissociated tumor cells were consecutively labeled with CD45 and CD133 magnetic MicroBeads at 4°C for 30 minutes each. Depletion of $CD45^+$ cells was followed by separation of $CD133^+$ and $CD133^–$ cell populations utilizing MACS LS columns (Miltenyi Biotec). All differential mRNA expression data were obtained from cells lysed immediately following magnetic sorting. Total RNA for RT-PCR was prepared using the NucleoSpin System (Macherey-Nagel) and complementary DNA transcribed using SuperScript II reverse transcriptase (Invitrogen). Complementary DNA amplification was monitored using SYBR Green Master Mix (Applichem) on the 7300 Real time PCR System (Applied Biosystems). The conditions for these PCR reactions were: 40 cycles of 95°C for 15 s, 60°C for 1 minute. The proper separation of $CD133^+$ and $CD133^-$ cells was confirmed by analyzing CD133 mRNA levels in the two cell populations. Arf1 transcript levels were used as a house-keeping reference for relative quantification of messenger RNA expression levels using the ΔCT method.

Flow cytometry

For detection of Grm3 and CD44 cell surface protein levels, GSC were separated mechanically and with accutase (Gibco Life Technologies, Paisley, UK) and incubated with anti-Grm3-Alexa647 antibody (1:100, bs-12012R-A647, BioSS/Lucerna, Lucerne, Switzerland), anti-CD44-FITC antibody (1:100, 11-0441-82, eBioscience, Vienna, Austria) or respective isotype controls, diluted in phosphate-buffered saline (PBS) containing 0.5% bovine serum albumin, 2 mM EDTA and 1 mM $MgCl_2$. Protein levels were determined using a CyAn flow cytometer (Beckman Coulter).

Immunohistochemistry

Frozen brains of pre-randomized mice embedded in cryomoulds in Shandon Cytochrome yellow (Thermo Scientific, Waltham, MA) were cut into 8 µM sections, fixed with 4 % paraformaldehyde and blocked with blocking solution (Biosystems, Muttenz, Switzerland). Sections were stained with primary antibodies to caspase-3 (#9662; Cell Signaling), cleaved caspase-3 (ASP175, #9661; Cell Signaling), Grm3 (1:200, bs-12012R, BioSS/Lucerna, Lucerne, Switzerland), Oct4 (1:200, ab184665, Abcam, Cambridge, UK) or Sox2 (1:300, ab171380, Abcam, Cambridge, UK) overnight at 4°C. Visualization was done using the SignalStain® Boost IHC Detection Reagent (HRP, Rabbit) (#8114; Cell Signaling) and the ImmPact DAB kit (#SK-4105, Vector Laboratories, Burlingame, CA).

Cyclic adenosine monophosphate assay

A cell-based competitive immunosorbent assay kit to detect cAMP was utilized (Cisbio). In brief, 5,000 LN-229 cells were washed twice with PBS without calcium or magnesium prior to adenyl cyclase stimulation with forskolin at 10 µM and addition of the Grm2/3 agonist LY-379268 at 100 nM or the Grm2/3 allosteric inhibitor RO1 or both. After 15 minutes the reaction was stopped by adding lysis buffer and detection reagents to initiate fluorescence resonance energy transfer (FRET) between XL665-labelled cAMP and a cryptate-labelled monoclonal anti-cAMP antibody. Fluorescent signals were detected at 620 nm and 665 nm, respectively, upon stimulation at 320 nm utilizing a Tecan infinite plate reader (Tecan, Maennedorf, Switzerland). Intercellular cAMP levels were calculated based on the reduction of FRET due to competition of cellular cAMP with XL665-labelled cAMP.

Immunoblot analysis

Denatured whole protein lysates (20 µg/lane) were separated on 10% acrylamide gels. After transfer to nitrocellulose (Bio-Rad), blots were blocked in PBS containing 5% skimmed milk and 0.05% Tween 20 and incubated overnight at 4°C with an anti-pERK antibody (Santa Cruz Biotechnology), washed in PBS and incubated for 1 h at room temperature with a horseradish peroxidase-coupled secondary antibody (Santa

Cruz Biotechnology) and the enhanced chemiluminescence technique (Thermo Fisher Scientific).

Clonogenic growth and sphere formation assays

For clonogenic growth assays of non-GSC, 500 cells per well were seeded in 6-well plates in triplicates overnight and exposed to temozolomide (TMZ) at the individual EC_{50} of each cell line [2] and/or to RO1 or RO2 at 100 nM for 24 h. After 21 days, colonies of at least 50 cells were counted upon staining with crystal violet. Sphere formation of GSC was assessed by separating neurospheres mechanically and with accutase, seeding overnight prior to treatment with RO1 or RO2 at indicated concentrations for 24 hours prior to seeding at 500 cells in 2 ml serum-free neurobasal medium supplemented with growth factors in uncoated culture dishes. Spheres of at least 50 cells were counted after 21 days. Susceptibility of clonogenic growth of GSC to co-treatment with RO1 or RO2 and TMZ was assessed by seeding 100 cells per well in 96-well plates and assessing cell viability after 7-14 days utilizing the MTT assay.

Note S1A

Glioblastoma gene expression subtypes were termed proneural, classical, mesenchymal and neural based on similarities with established gene set expression patterns.[3] The neural subtype reflects mostly normal brain expression patterns in samples with low tumor cell content, e.g. in the infiltration zone of tumors.[4, 5] Proneural glioblastomas comprise a sub-group of tumors that harbor distinct point mutations in the genes encoding isocitrate dehydrogenase (*IDH*)-1 or -2.[3] Patients with *IDH* mutated glioblastomas are younger and experience a relatively more favourable outcome.[6] Therefore, we considered that imbalances in *GRM3* gene expression between *IDH* mutated and *IDH* wild type proneural glioblastomas may explain the association of *GRM3* expression levels with survival. *GRM3* gene expression was balanced between both groups (t-test p=0.93, Fig. S1D) and the transcriptional subtypes do not differ in terms of survival,[3] including when analysis is restricted to patients with IDH wild-type glioblastoma.[4] At an alpha level of 0.1, the association of *GRM3* expression levels with survival was also apparent in the proneural group when patients with IDH mutated glioblastoma were excluded (Fig. S1E, p=0.089). No association of *GRM3* gene expression levels with OS was detected in IDH mutated proneural glioblastomas, albeit the small number of only 9 patients per group precludes definite conclusions (Fig. S1F, p=0.78). No association of high versus low *GRM3* expression levels with overall survival was present in patients with lower-grade gliomas (not shown, log rank p=0.41), which comprised mostly IDH mutated astrocytomas and oligodendrogliomas.[7]

Note S1B

We considered chromosome 7 amplification a likely explanation for high Grm3 expression, because chromosome 7 gain is frequent in IDH wild-type glioblastoma and underlies the upregulation of several oncogenes.[6, 8-10] Indeed, copy number gains of the Grms located on chromosome 7, i.e. *GRM3* and *GRM8*, are present in >75% of glioblastoma samples (Fig. S1G), but *GRM3* copy number gains appear not to be associated with gene expression (Fig. S1H). Therefore, we next interrogated gene methylation patterns of *GRM* genes. The gene encoding Grm3 was the least methylated among all Grm subtypes (Fig. S1I). Overall, the mechanism of differential regulation of *GRM3* remains elusive.

Supplementary Figures

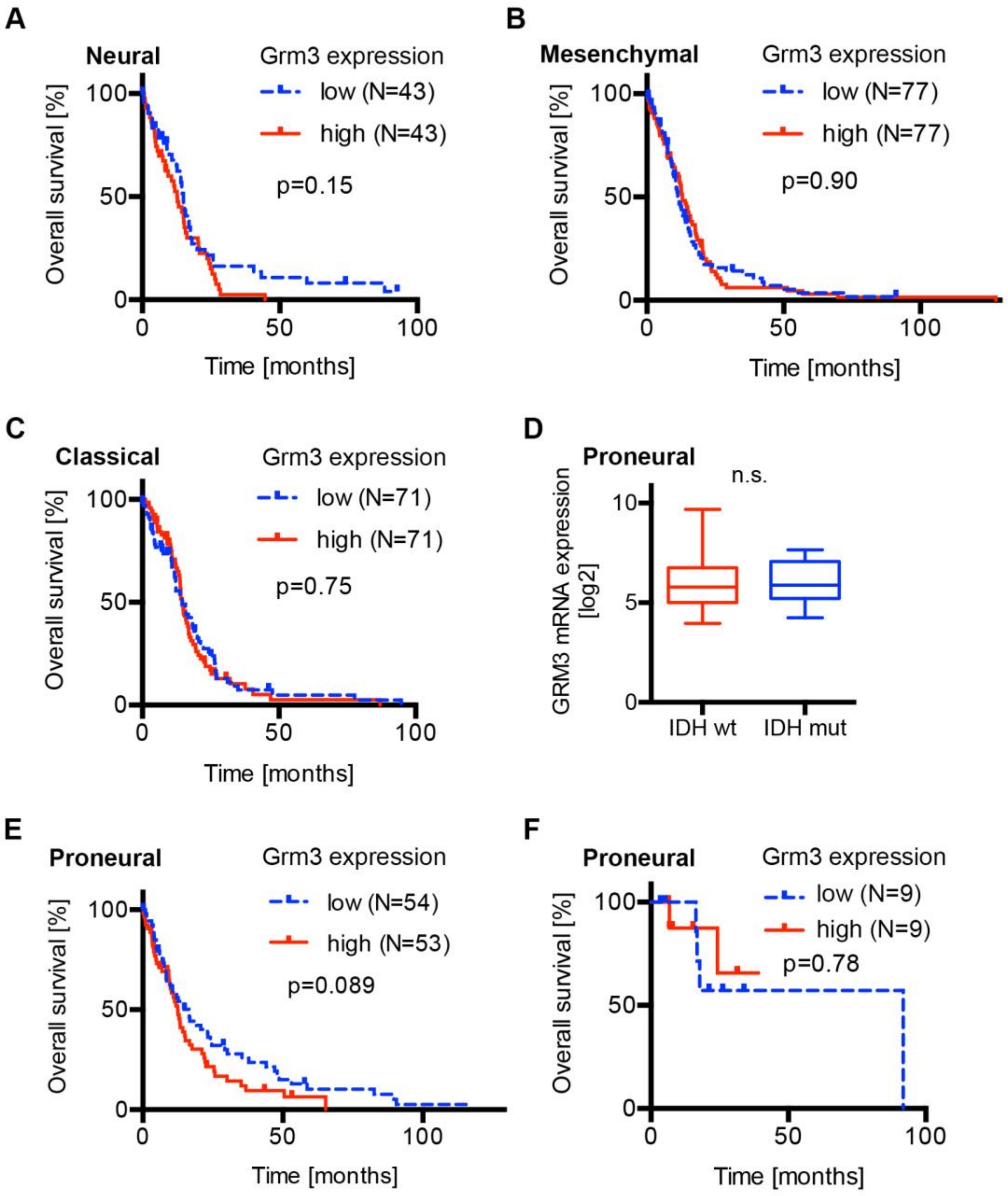

(Figure S1, continued on next page)

(Figure S1, continued)

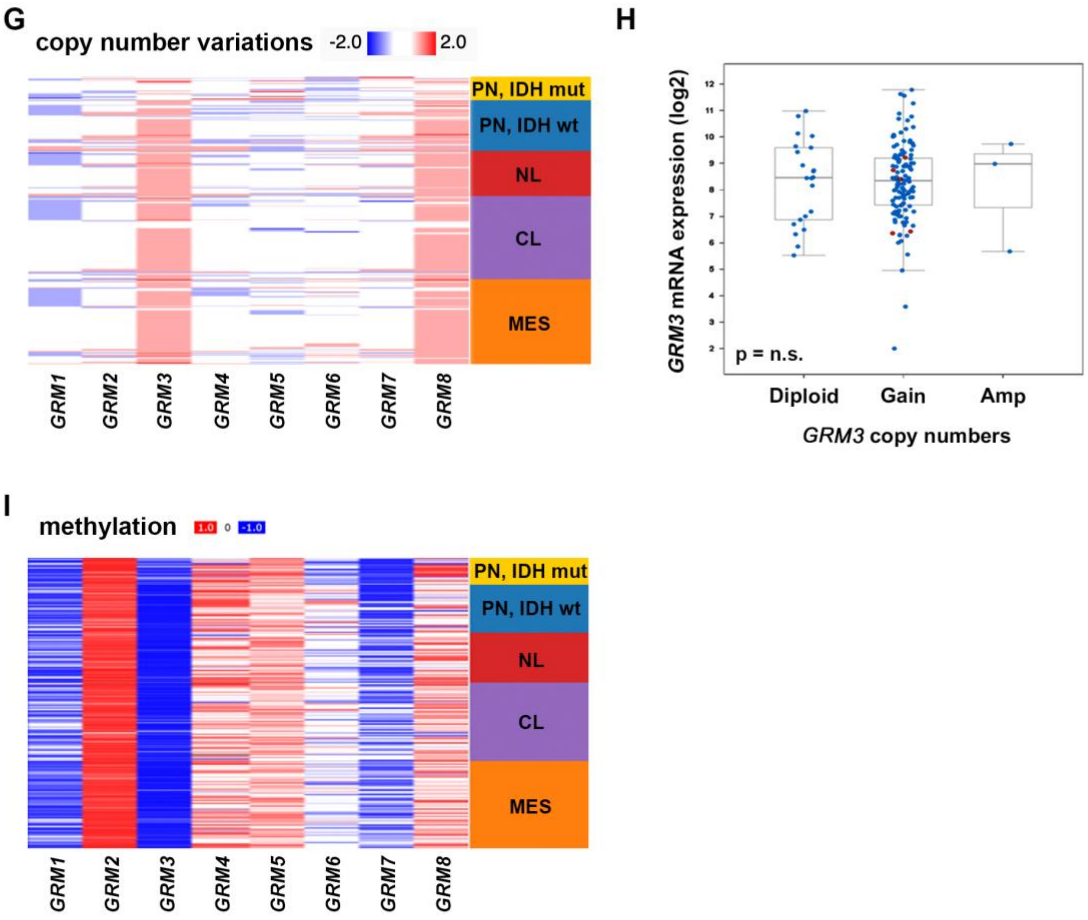

Figure S1. Outcome and molecular analyses of the TCGA glioblastoma dataset. *A-C.* Kaplan Meier curve of patients with indicated glioblastoma gene expression subtypes, stratified by *GRM3* gene expression levels (cut-off: median *GRM3* gene expression). *D. GRM3* gene expression in proneural glioblastoma with IDH wild-type (N=105) versus mutant (N=18); Boxplots represent median and interquartile range, whiskers represent the range; n.s., not significant. *E,F.* overall survival stratified by low versus high *GRM3* gene expression (cut-off: median) in patients with proneural glioblastoma with wild-type *(E)* and mutant *(F)* IDH. *G.* Copy number variations of *GRM* genes by gene expression subtype. Copy numbers were determined by GISTIC (thresholded) utilizing the Xena webtool.[11] *H. GRM3* mRNA expression by copy number. Boxplots represent median and interquartile range (n.s., not significant). *I.* Gene methylation patterns of *GRM* genes (N=288).

A

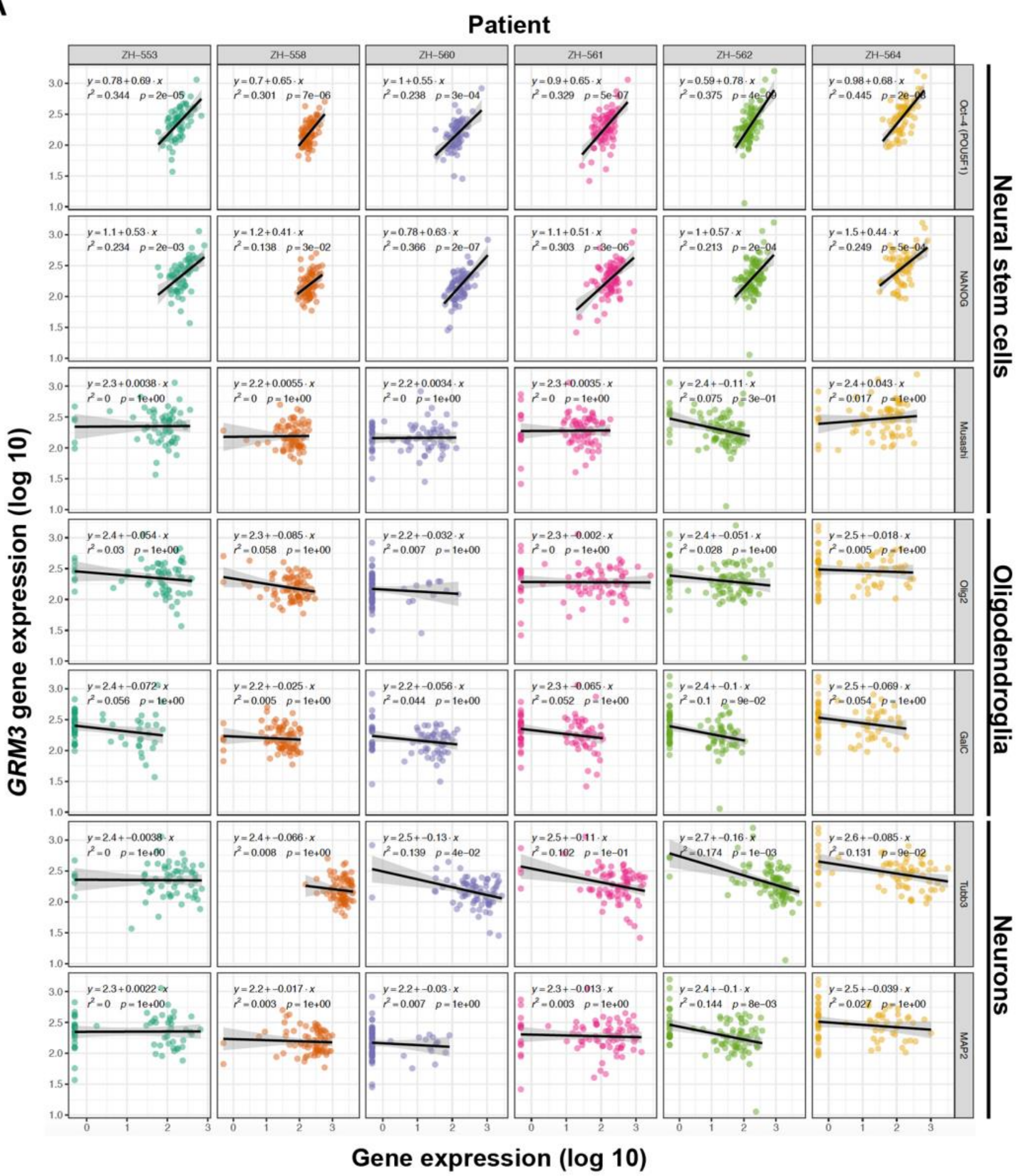

(Figure S2, continued on next page)

(Figure S2, continued)

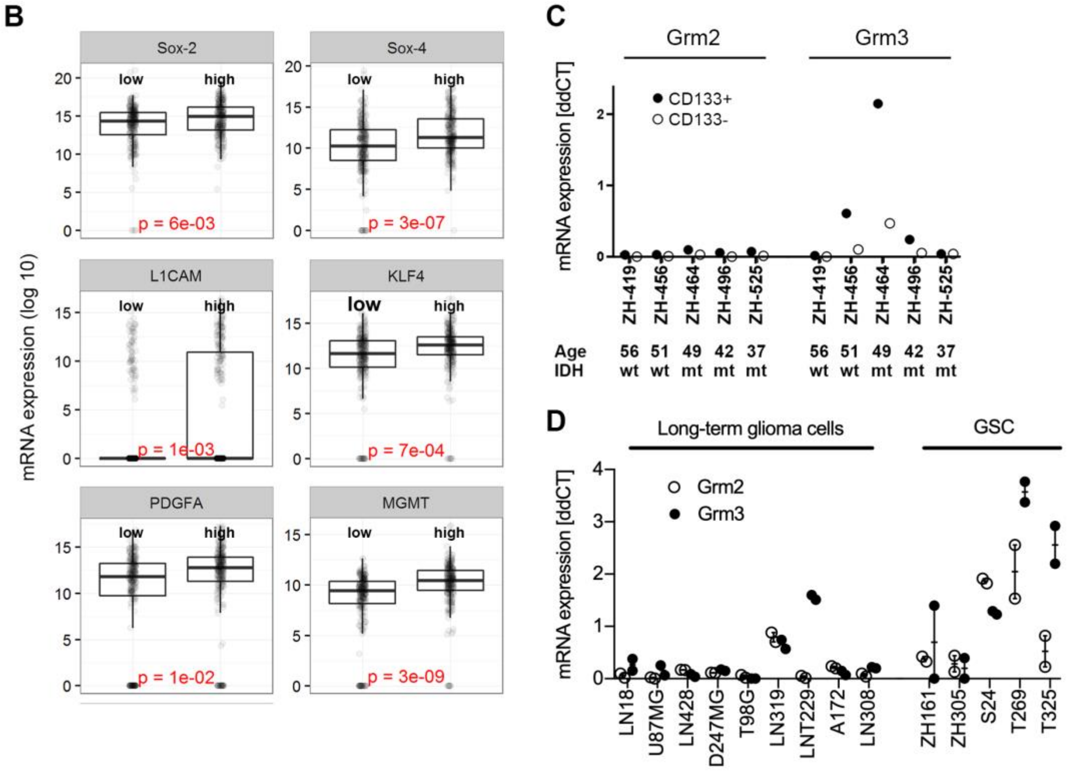

Fig. S2. Grm3 expression in single cells, GSC and cell line models. *A, B.* Single cell gene expression analysis in freshly dissected glioblastoma samples (N=6). Gene expression values of individual cells were obtained by scRT-PCR and cell-to-cell median normalization. *A.* Co-expression of Grm3 and neuroglial lineage markers in individual glioblastoma cells *ex vivo*. Individual patients are color-coded and referenced in the top line. Grm3 gene expression is plotted on the y-axis, genes analysed are indicated on the right and plotted on the x-axis. Slopes indicate correlations. Black line indicates a linear regression fitted to the data in each panel; grey area shows 95% confidence interval of this fit; text shows the formula of the fitted linear regression, the R^2 value and the Bonferroni-adjusted p-value for the fit. *B.* Gene expression in single glioblastoma cells with low versus high Grm3 expression. Data from individual patients were pooled (N=6) and cells were stratified by low versus high *GRM3* gene expression (N=482, cut-off: median). Boxes depict the median and interquartile ranges, whiskers extend to the largest value no further than 1.5-fold interquartile ranges from the hinge. Two-sided t-test and Bonferroni adjustment for multiple testing was performed to compare gene expression in low versus high Grm3 expressing cells. *C.* RT-PCR of Grm2 and Grm3 mRNA levels in freshly dissected tumor tissue (N=5) sorted for CD133. *D. GRM2* and *GRM3* gene expression in a panel of long-term glioma cell lines and GSC (mean and SEM, n=2).

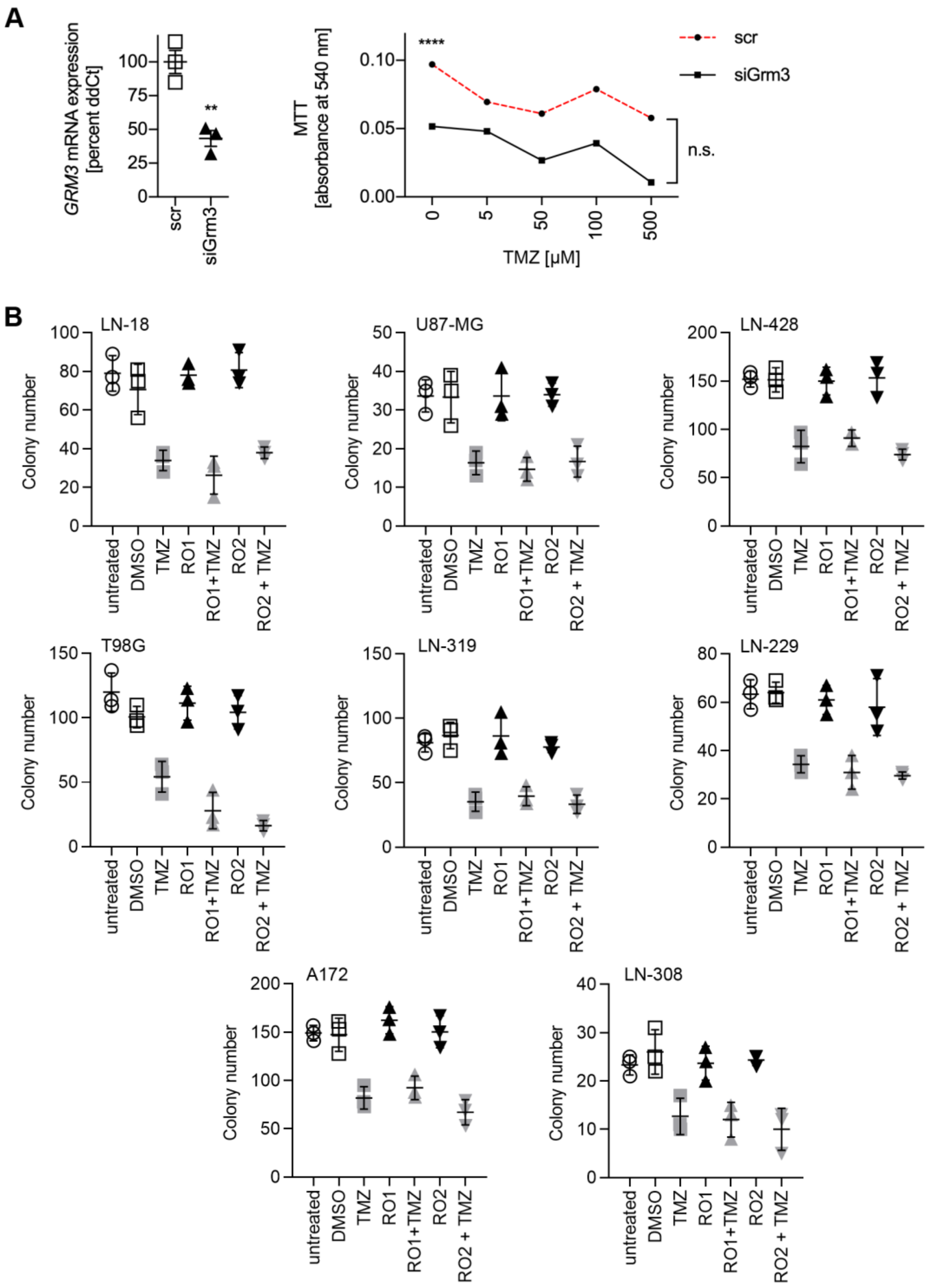

Fig. S3. Inhibition of Grm3 signalling in non-GSC does not affect clonogenic growth or sensitivity to temozolomide (TMZ). *A.* 72 h after transfection of T-325 cells with *GRM3*-targeted siRNA, gene silencing was confirmed by qRT-PCR (left panel), and 100 T-325 cells per well were seeded in 96-well plates in 6 technical replicates and metabolic activity was assessed utilizing the MTT assay on day 7 **** p<0.001, t-test corrected for multiple testing utilizing the Holms-Sidak method, error bars: SEM). *B.* Indicated cell lines were seeded at clonal density (500 cells per well in 6-well plates) and treated overnight with TMZ at

the individual EC_{50} of each cell line [2], Grm3 signalling inhibitors (RO1, RO2) at 100 nM, or both. Dimethylsulfoxide (DMSO) was utilized as solvent control. Colony numbers were counted after 21 days. 2-sided t-tests comparing between RO1 or RO2 versus DMSO and RO1+TMZ or RO2+TMZ versus TMZ were not significant for any cell line tested.

Table S1. Clinical and molecular characteristics of patients' samples utilized for single cell RT-PCR.

**Patient ID**	**Number of cells**	**Age/gender**	**Diagnosis**	**MGMT**	**EGFRvIII**
ZH-553	69	63/f	Glioblastoma, IDH wildtype	methylated	yes
ZH-558	82	29/f	Glioblastoma, IDH wildtype	not methylated	No
ZH-560	78	54/m	Glioblastoma, IDH wildtype	not methylated	No
ZH-561	84	33/f	Glioblastoma, IDH wildtype	methylated	Yes
ZH-562	94	82/m	Glioblastoma, IDH wildtype	not methylated	No
ZH-564	75	54/m	Glioblastoma, IDH wildtype	not methylated	No

Supplementary References

1. Livak, KJ, Wills, QF, Tipping, AJ, Datta, K, Mittal, R, Goldson, AJ*, et al.* (2013). Methods for qPCR gene expression profiling applied to 1440 lymphoblastoid single cells. *Methods* **59**: 71-79.

2. Hermisson, M, Klumpp, A, Wick, W, Wischhusen, J, Nagel, G, Roos, W*, et al.* (2006). O6-methylguanine DNA methyltransferase and p53 status predict temozolomide sensitivity in human malignant glioma cells. *J Neurochem* **96**: 766-776.

3. Verhaak, RG, Hoadley, KA, Purdom, E, Wang, V, Qi, Y, Wilkerson, MD*, et al.* (2010). Integrated genomic analysis identifies clinically relevant subtypes of glioblastoma characterized by abnormalities in PDGFRA, IDH1, EGFR, and NF1. *Cancer cell* **17**: 98-110.

4. Wang, Q, Hu, B, Hu, X, Kim, H, Squatrito, M, Scarpace, L*, et al.* (2018). Tumor Evolution of Glioma-Intrinsic Gene Expression Subtypes Associates with Immunological Changes in the Microenvironment. *Cancer cell* **33**: 152.

5. Reifenberger, G, Weber, RG, Riehmer, V, Kaulich, K, Willscher, E, Wirth, H*, et al.* (2014). Molecular characterization of long-term survivors of glioblastoma using genome- and transcriptome-wide profiling. *Int J Cancer* **135**: 1822-1831.

6. Reifenberger, G, Wirsching, HG, Knobbe-Thomsen, CB, and Weller, M (2017). Advances in the molecular genetics of gliomas - implications for classification and therapy. *Nat Rev Clin Oncol* **14**: 434-452.

7. Ceccarelli, M, Barthel, FP, Malta, TM, Sabedot, TS, Salama, SR, Murray, BA, *et al.* (2016). Molecular Profiling Reveals Biologically Discrete Subsets and Pathways of Progression in Diffuse Glioma. *Cell* **164**: 550-563.

8. Cancer Genome Atlas Research, N (2008). Comprehensive genomic characterization defines human glioblastoma genes and core pathways. *Nature* **455**: 1061-1068.

9. Ozawa, T, Riester, M, Cheng, YK, Huse, JT, Squatrito, M, Helmy, K*, et al.* (2014). Most human non-GCIMP glioblastoma subtypes evolve from a common proneural-like precursor glioma. *Cancer cell* **26**: 288-300.

10. Cimino, PJ, Kim, Y, Wu, HJ, Alexander, J, Wirsching, HG, Szulzewsky, F*, et al.* (2018). Increased HOXA5 expression provides a selective advantage for gain of whole chromosome 7 in IDH wild-type glioblastoma. *Genes Dev* **32**: 512-523.

11. Goldman, M, Craft, B, Hastie, M, Repečka, K, Kamath, A, McDade, F*, et al.* (2019). The UCSC Xena platform for public and private cancer genomics data visualization and interpretation. *bioRxiv*: 326470.
